# uPA-mediated remodeling of CCL21 gradients regulates lymphatic migration of dendritic cells

**DOI:** 10.1083/jcb.202412190

**Published:** 2026-01-27

**Authors:** Victor Collado-Diaz, Maria-Nefeli Christakopoulou, Philipp Schineis, Katharina Blatter, David Laubender, Sébastien Trzebanski, Marina Thoma, Yves Gadient, Hazal Tatliadim, Konstantinos Gkelis, Mona C. Friess, Vladimir Purvanov, Guerric P.B. Samson, Marc Artinger, Radjesh Bisoendial, Manuel Yepes, Simon J. de Veer, David Craik, Niels Behrendt, Karina Silina, Daniel F. Legler, Cornelia Halin

**Affiliations:** 1 Institute of Pharmaceutical Sciences, ETH Zurich, Zurich, Switzerland; 2 Department of Pharmacology, University of Valencia, Valencia, Spain; 3 Max Planck Institute for Biological Intelligence, Martinsried, Germany; 4 Institute of Cell Biology and Immunology Thurgau (BITG) at the University of Konstanz, Kreuzlingen, Switzerland; 5 Department of Rheumatology and Clinical Immunology, Maasstad Hospital, Rotterdam, The Netherlands; 6 Department of Immunology, Erasmus Medical Center, Rotterdam, The Netherlands; 7 Department of Neurology, Emory University, Atlanta, GA, USA; 8 Institute for Molecular Bioscience, Australian Research Council Centre of Excellence for Innovations in Peptide and Protein Science, The University of Queensland, Brisbane, Australia; 9 Finsen Laboratory, Rigshospitalet/Biotech Research and Innovation Centre (BRIC), University of Copenhagen, Copenhagen, Denmark; 10 Theodor Kocher Institute, University of Bern, Bern, Switzerland; 11 Faculty of Biology, University of Konstanz, Konstanz, Germany

## Abstract

Dendritic cell (DC) migration via afferent lymphatics to draining LNs (dLNs) occurs in distinct steps that require the chemokine C–C motif ligand 21 (CCL21). In addition to full-length CCL21, which forms an immobilized perilymphatic gradient, a truncated soluble variant with enhanced gradient-forming capacity (CCL21-ΔC) was recently identified in tissues. We show that in skin, plasmin is continuously activated in a urokinase plasminogen activator (uPA)-dependent manner on lymphatic endothelial cells (LECs) and cleaves full-length CCL21, generating CCL21-ΔC. Inflammatory conditions, while promoting overall DC migration, markedly enhance this process, reducing immobilized perilymphatic CCL21 and increasing dermal CCL21-ΔC levels. Inhibition of uPA-mediated CCL21 cleavage causes full-length CCL21 to accumulate around dermal lymphatics, while CCL21-ΔC levels decline in the skin and dLN subcapsular sinus. Consequently, DC entry into afferent lymphatics is diminished, whereas DC egress from the subcapsular sinus into the LN parenchyma is enhanced. These findings reveal uPA/plasmin-dependent regulation of lymphatic CCL21 gradients and identify CCL21-ΔC as critical for DC migration.

## Teaser

Collado-Diaz et al., show that lymphatic endothelial cells generate a soluble chemokine variant, CCL21-ΔC, through uPA/plasmin-dependent cleavage. This process reshapes chemokine gradients in skin and LNs, thereby regulating dendritic cell migration and positioning and uncovering a proteolytic mechanism that fine-tunes immune surveillance.

## Introduction

**Figure 1.**
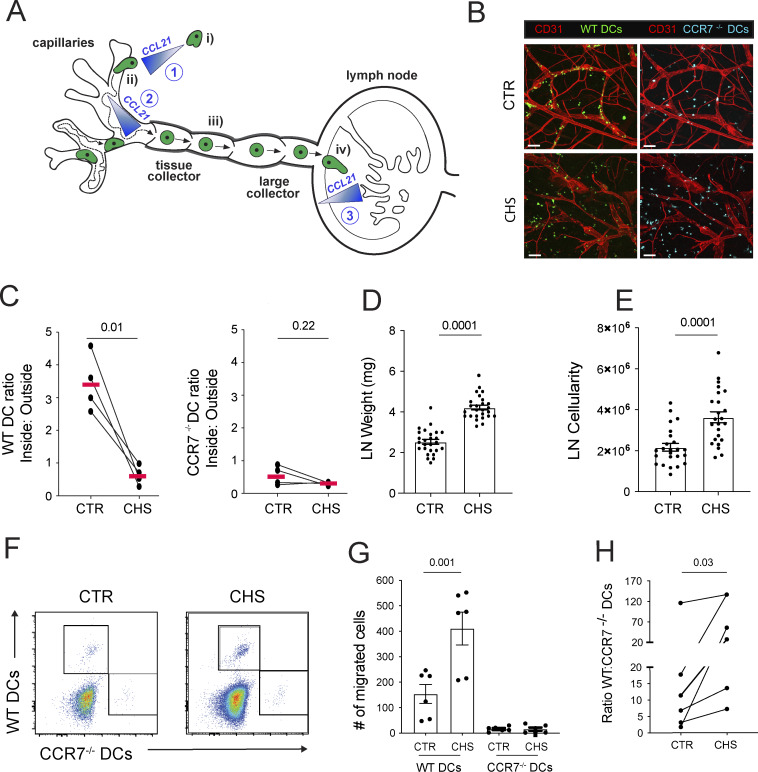
**CHS-induced dermal inflammation impairs DC entry into lymphatics in *in vitro* crawl-in assays but enhances their *in vivo* migration. (A)** Current model of the different steps in DC migration from skin to dLNs and their documented dependence on CCL21 gradients. DCs approach dermal lymphatics migrating along a perilymphatic CCL21 gradient (1). Upon entry into lymphatic capillaries (i), DCs actively migrate in a semidirected manner, following the immobilized CCL21 gradient (2) deposited in the capillary lumen (ii). Once lymph flow picks up due to LV contractions in collectors, DCs are passively transported (iii) to the LN SCS. Egress from the SCS into the LN parenchyma (iv) occurs along another CCL21 gradient (3) **(B and C)** Crawl-in assay: Mice were sensitized with 2% oxazolone on the belly on day 0 and challenged on day 5 by applying 1% oxazolone to the skin of one ear. Crawl-in assays with both ears, i.e., the CTR and the CHS-inflamed ear, were performed 1 day later by adding fluorescently labelled LPS-matured WT and CCR7^−/−^ bone marrow–derived DCs (1:1 ratio) onto the dermal ear skin to allow DCs to migrate into lymphatics for 4 h. (B) Representative images of WT and CCR7^−/−^ bone marrow–derived DCs and the vasculature (stained for CD31) at the end of the experiment. Scale bar: 100 μm. (C) Quantification of the ratio of WT (left) and CCR7^−/−^ (right) bone marrow–derived DCs located inside vs. outside of lymphatics. *n* = 4 experiments performed with 1 mouse each. Paired Student’s *t* test. **(D–H)***In vivo* DC migration assay: 1:1 mixture of LPS-matured and CCR7^−/−^ DCs, labelled in two fluorescent colors, were transferred into either CHS-inflamed or uninflamed CTR footpads. DC numbers in draining popliteal LNs were quantified by flow cytometry 16–18 h later. **(D)** Popliteal LN weight. **(E)** Popliteal LN cellularity. **(F)** Gating scheme used for the identification of transferred DCs. **(G)** Quantification of DC numbers in popliteal LNs. Pooled data from six experiments are shown (total *n* = 25 CTR and *n* = 25 CHS). **(H)** Ratio of migrated WT:CCR7^−/−^ DCs per experiment. Statistics: unpaired (D, E, and F) and paired (H—since in same animal) Student’s *t* test.

The migration of antigen-presenting dendritic cells (DCs) from peripheral tissues to draining LNs (dLNs) is important for the induction of adaptive immunity and for the maintenance of tolerance (Bauer et al., 2022; Permanyer et al., 2018). Although DCs continuously migrate to dLNs in steady-state, their migration is strikingly enhanced during inflammation or infection (Ohl et al., 2004; Vigl et al., 2011). Research over the past two decades has revealed that DC migration via afferent lymphatic vessels (LVs) occurs in distinct steps, which all highly depend on the chemokine C–C motif ligand 21 (CCL21) and its DC-expressed receptor C–C chemokine receptor type 7 (CCR7) (Arasa et al., 2021a; Förster et al., 1999; Permanyer et al., 2018) (Fig. 1 A). In peripheral tissues, CCL21 is exclusively produced by the lymphatic endothelial cells (LECs) and accumulates in vesicles of the trans-Golgi network from where it is secreted into the surrounding tissue (Weber et al., 2013). Upon secretion, CCL21’s highly positively charged C terminus immobilizes the chemokine on the negatively charged LEC glycocalyx or in the surrounding extracellular matrix (Bao et al., 2010; Vaahtomeri et al., 2017). This immobilization establishes a perilymphatic CCL21 gradient, which radiates up to 50 µm into the tissue. In skin explants, this gradient was shown to guide haptotactic migration of CCR7-expressing DCs toward the lymphatic vasculature (Weber et al., 2013). Besides attracting DCs toward lymphatics, CCL21 supports several other steps of lymphatic migration (Fig. 1 A), such as the docking of DCs to LECs of lymphatic capillaries (Tal et al., 2011) and guidance to LEC junctions (Liaqat et al., 2024), thereby initiating their transmigration into the capillary lumen (Lämmermann et al., 2008; Pflicke and Sixt, 2009).

Once within lymphatic capillaries, DCs need to actively crawl and migrate, as the flow in this compartment is too weak to sustain passive transport (Fig. 1 A). Intralymphatic DC crawling occurs in a semi-directed manner, following a flow-deposited, downstream-oriented intralymphatic CCL21 gradient that guides DCs into the direction of the collecting vessel segments (Russo et al., 2016). Here, flow increases due to LV contractions, allowing for DC de-adhesion and rapid, flow-mediated transport to the dLNs. Upon arrival in the LN subcapsular sinus (SCS) (Fig. 1 A), DCs sense yet another CCL21 gradient that guides them from the SCS into the CCL21-rich LN parenchyma (Ulvmar et al., 2014). The latter is established by the atypical chemokine receptor 4 (ACKR4), which is expressed in the LECs forming the ceiling of the SCS and scavenges CCL21 for internalization and degradation (Ulvmar et al., 2014).

In addition to full-length CCL21 forming these well-described immobilized gradients, emerging findings have revealed that CCL21 also exists in a cleaved form called CCL21 with truncated C terminus (CCL21-ΔC). The cleaved form lacks the 32–amino acid positively charged C terminus of full-length CCL21, is more soluble compared with full-length CCL21, and displays enhanced DC chemotactic activity at lower chemokine concentrations (Lorenz et al., 2016; Moussouras et al., 2020; Schumann et al., 2010). Originally, an *in vitro* study reported that DCs themselves can generate CCL21-ΔC, as demonstrated with recombinant full-length CCL21 or endogenous CCL21 present in LN tissue slices (Schumann et al., 2010). Only recently, the existence of CCL21-ΔC was also reported *in vivo* in the skin and in several other peripheral murine tissues (Bastow et al., 2021). Both endogenous full-length CCL21 and CCL21-ΔC were found to be scavenged by ACKR4, which in the skin is expressed in some fibroblasts, LECs, as well as in keratinocytes (Bastow et al., 2021). In ACKR4-deficient mice, CCL21-ΔC levels were increased in various peripheral tissues. Moreover, the immobilized perilymphatic CCL21 gradient was saturated, resulting in less directed migration and reduced DC entry into dermal lymphatics (Bastow et al., 2021). However, how CCL21-ΔC is generated *in vivo* and whether both variants work together in supporting DC migration *in vivo* is presently not known. Although the original report on CCL21 cleavage by DCs had indicated the contribution of a DC-expressed serine protease in this process, its precise identity remained unknown (Schumann et al., 2010). Other *in vitro* studies subsequently reported that human plasmin could cleave human CCL21 to CCL21-ΔC (Loef et al., 2022; Lorenz et al., 2016). The serine protease plasmin is well-known for its vital role in fibrinolysis and is generated *in vivo* from plasminogen through proteolytic cleavage mediated by either the tissue or the urokinase plasminogen activator (tPA and uPA). The latter serine proteases are expressed by numerous stromal and hematopoietic cells (Ismail et al., 2021; Urano et al., 2018). Conversely, plasminogen is exclusively produced in the liver and is present at high levels in blood (Deryugina and Quigley, 2012).

This study was sparked by our unexpected finding that the immobilized dermal CCL21 gradient was markedly reduced during skin inflammation, while migration of adoptively transferred DCs from inflamed skin to dLNs was significantly enhanced. We show that this paradox is explained by CCL21 cleavage, which continuously occurs in steady-state skin and is strikingly increased under inflammatory conditions. We further demonstrate that CCL21 cleavage is plasmin/uPA/uPA receptor (uPAR) dependent and that the resulting soluble and immobilized CCL21 gradients jointly fine-tune DC migration.

## Results

### Contact hypersensitivity–induced inflammation impairs DC entry into lymphatics in *in vitro* crawl-in assays but enhances their *in vivo* migration

The role of the immobilized perilymphatic CCL21 gradient in guiding DC migration toward lymphatics has mainly been studied using so-called crawl-in assays, where lipopolysaccharide (LPS)-matured, CCR7^hi^ bone marrow–derived DCs are applied *in vitro* to the dermal aspects of steady-state murine ear skin, leading to their migration along the immobilized CCL21 gradient into dermal lymphatics (Arasa et al., 2021b; Bastow et al., 2021; Holtkamp et al., 2021; Pflicke and Sixt, 2009; Weber et al., 2013). In contrast to this assay, CCR7^hi^ DCs *in vivo* primarily migrate in the context of tissue inflammation/infection, when DCs are matured by pathogen- or danger-associated molecular patterns (Bauer et al., 2022; Permanyer et al., 2018). To better reflect this physiological context, we performed crawl-in assays in murine ear skin that had been inflamed by inducing a contact hypersensitivity (CHS) response. CHS is a T cell–mediated inflammatory reaction triggered by sensitizing mice to a hapten (e.g., oxazolone) on the belly and paws, followed by challenge on the ear skin 5 days later, to elicit local T cell–mediated inflammation (Honda et al., 2013). Surprisingly, in the crawl-in assay, migration of fluorescent WT DCs into LVs was profoundly reduced when applied to CHS-inflamed skin compared with steady-state control (CTR) ear skin (Fig. 1, B and C). As expected, fluorescent CCR7^−/−^ DCs, which were simultaneously added, failed to migrate under either condition (Fig. 1, B and C). Since DC migration to dLNs is well established to increase under inflammatory conditions (MartIn-Fontecha et al., 2003; Ohl et al., 2004; Vigl et al., 2011), we wanted to exclude the possibility that the reduced lymphatic entry observed in inflamed skin was due to compromised DC quality. To this end, we performed a competitive adoptive transfer by injecting equal numbers of LPS-matured, fluorescently labelled WT and CCR7^−/−^ bone marrow–derived DCs into CTR or CHS-inflamed footpads and analyzed their numbers in popliteal dLNs 24 h later. LN weight and cellularity were increased in LNs draining CHS-inflamed footpads (Fig. 1, D and E). Consistent with the literature, WT DC migration to popliteal dLNs was significantly enhanced under inflammatory conditions (Fig. 1, F–G), confirming the functional quality of our DCs. However, in contrast to the crawl-in results (Fig. 1, B and C), *in vivo* DC migration was even more CCR7-dependent under CHS than CTR conditions (Fig. 1 H). Moreover, performing intravital microscopy in CTR or CHS-inflamed ear skin, we consistently detected more endogenous DCs inside of or colocalizing with inflamed dermal lymphatics than in CTR conditions (Videos 1 and 2). Thus, the enhanced DC migration observed in CHS-inflamed skin *in vivo* (Fig. 1 G) was not faithfully mimicked when performing the *in vitro* crawl-in assay in CHS-inflamed skin explants (Fig. 1, B and C).

**Video 1.** **Video showing DCs crawling around or inside LVs in uninflamed murine ear skin.** Intravital microscopy was performed on uninflamed ear skin of anesthetized Prox1-mOrange YFP-CD11c mouse. DCs (green) can be observed crawling around or inside LVs (red). The video was generated from 3D reconstructions of Z-stacks. Video specifications: 20× objective, 30-s intervals, and 10 frames/s (300-fold accelerated). The original length of the recording: 60 min. Scale bar, 50 μm. Video length: 16 s.

**Video 2.** **Video showing DCs crawling around or inside LVs in inflamed murine ear skin.** Intravital microscopy was performed on CHS-inflamed ear skin of anesthetized Prox1-mOrange YFP-CD11c mouse. DCs (green) can be observed crawling around or inside LVs (red). The video was generated from 3D reconstructions of Z-stacks. Video specifications: 10× objective, 30-s intervals, and 10 frames/s (300-fold accelerated). The original length of the recording: 60 min. Scale bar, 100 μm. Video length: 16 s.

### Immobilized, perilymphatic CCL21 is diminished in CHS-inflamed skin, while more CCL21 is secreted from LEC intracellular stores

**Figure 2.**
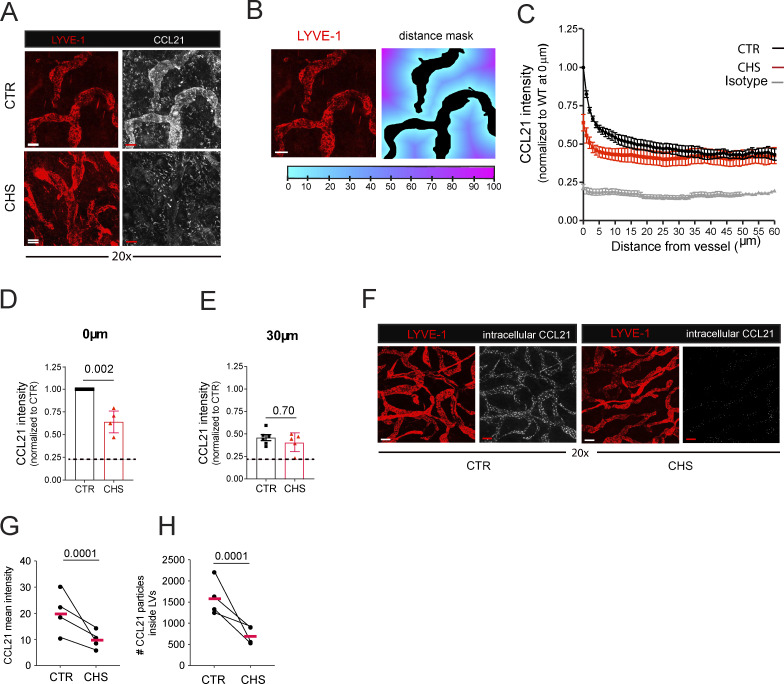
**The immobilized perilymphatic CCL21 gradient is diminished during CHS-induced skin inflammation. (A–E)** Analysis of the immobilized perilymphatic CCL21 gradient stained in fresh (unfixed) CTR and CHS-inflamed ear skin (24 h after challenge). **(A)** Representative images showing the CCL21 and LYVE-1 signal at 20× magnification (scale bar: 50 μm). **(B)** Based on the LYVE-1 signal, a mask was generated to analyze the CCL21 intensity in relation to distance from the nearest LV. Scale bar: 100 μm. **(C–E)** Quantification of the CCL21 staining intensity as a function of the distance from the nearest LYVE-1^+^ LV. CCL21 staining intensity was measured at (D) 0 μm or (E) 30 μm from the LV. *n* = 6 mice per condition (4–6 images analyzed per mouse). The dotted horizontal line in D and E indicates the level of background (isotype) staining. **(F–H)** Analysis of intracellular CCL21 deposits, revealed by staining in PFA-fixed CTR and CHS-inflamed ear skin. **(F)** Representative images. Scale bar: 50 μm. Quantification of G the intracellular CCL21 staining intensity and (H) the number of CCL21 deposits present within lymphatics. Pooled data from *n* = 4 mice/condition with 3–6 images/ear skin are shown. Data from the same experiment (i.e., same mouse) in G and H are connected by a line and analyzed by paired Student’s *t* test.

Since DC migration toward lymphatics highly depends on CCL21, we investigated the immobilized extracellular perilymphatic CCL21 gradient by performing whole-mount immunofluorescence in fresh, non-permeabilized CTR and CHS-inflamed ear skin (Fig. 2, A–E). To quantify the CCL21 signal dependent on the distance from the LV, we generated vessel masks based on staining for the lymphatic marker LV endothelial hyaluronan receptor 1 (LYVE-1) (Fig. 2 B). This analysis revealed that immobilized perilymphatic CCL21 levels were markedly diminished in CHS-inflamed skin compared with CTR skin (Fig. 2 C). This decrease in CCL21 signal was particularly pronounced in immediate proximity of the lymphatics (Fig. 2 D), whereas no significant differences were observed at distances >30 μm from the LV (Fig. 2 E). It was previously reported that treatment of cultured LECs or skin explants with the proinflammatory cytokine TNF-α (Johnson and Jackson, 2010), or cellular contact between LECs and DCs (Vaahtomeri et al., 2017), enhances the secretion of CCL21 from intracellular stores in the trans-Golgi network. When PFA fixing and permeabilizing the tissue—a method that primarily visualize the large and intensively stained intracellular CCL21 deposits present in LECs (Fig. 2 F)—we found that both the number and the staining intensity of intracellular CCL21 deposits were significantly reduced in lymphatics present in CHS-inflamed compared with CTR skin (Fig. 2, G and H), indicating increased CCL21 secretion under inflammatory conditions.

### A soluble CCL21 proteoform, CCL21-ΔC, exists and is increased in CHS-inflamed skin

**Figure 3.**
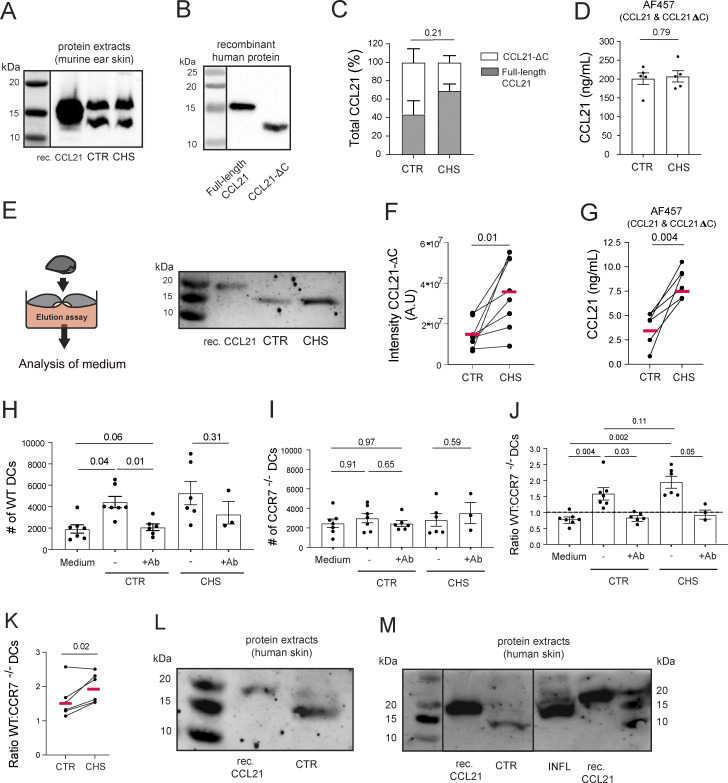
**A soluble proteoform of CCL21 (CCL21-ΔC) with chemotactic activity is present in murine skin and increased in CHS-inflamed skin. (A)** Representative western blot of CCL21 performed on steady-state (CTR) and CHS-inflamed (CHS) murine ear skin protein extracts. Recombinant CCL21 was loaded as a CTR. **(B)** Western blot analysis of recombinant human full-length CCL21 and CCL21-ΔC protein. One out of two experiments is shown. **(C)** Quantification of the full-length CCL21 (gray) and CCL21-ΔC (white) relative band percentages. Pooled data from *n* = 4 independent biological replicates. **(D)** ELISA-based quantification of total CCL21 in tissue protein extracts, performed with antibody clone AF457, which detects full-length CCL21 and CCL21-ΔC. Pooled data from *n* = 5 mice/condition. Statistics: unpaired Student’s *t* test. **(E–J)** Skin elution assay and analyses were performed on the supernatants. (E) Schematic depiction of the assay and representative western blot analysis. (F) Image-based quantification of the CCL21-ΔC band intensity from western blots as in E. A.U., arbitrary units, as produced by the western blot imager (G) ELISA-based quantification of total CCL21, performed with antibody clone AF457. Pooled data from *n* = 6–7 mice/condition, with one CHS-inflamed and a contralateral CTR ear, are shown in E and F. Data from the same mouse are connected by a line. The mean is shown in red, paired Student’s *t* test. (H–J) Transwell chemotaxis assays were performed on elution assay supernatants (see E) with 1:1 mixtures of LPS-matured labelled WT and CCR7^−/−^ DCs in presence/absence of a CCL21-blocking antibody. Flow cytometry–based quantification of the total numbers of transmigrated (H) WT DCs and (I) CCR7^−/−^ DCs, as well as of (J) the ratio of transmigrated WT to CCR7^−/−^ DCs. Data points from 3–7 experiments per condition are shown. Mixed effects statistical analysis. **(K)** Ratio of WT: CCR7^−/−^ DCs measured in seven paired experiments performed for CTR and CHS-inflamed condition. Statistical analysis: paired Student's *t* test. **(L and M)** Western blot analysis of protein extracts of (L) steady-state human skin (CTR) and (M) donor-matched steady-state (CTR) and inflamed (INF) human skin from a psoriasis patient. Data from one out of two experiments in L and one experiment in M are shown. Recombinant human full-length CCL21 was loaded as a CTR. Source data are available for this figure: SourceData F3.

Our results indicated that more CCL21 was released from intracellular stores during inflammation (Fig. 2, F–H), while at the same time, the immobilized perilymphatic CCL21 levels were diminished (Fig. 2, A–E). To resolve this apparent discrepancy, we performed western blot analysis of tissue protein extracts of CTR and CHS-inflamed ear skin, which revealed two bands of CCL21 of approximately ∼15 and ∼11 kDa, respectively (Fig. 3 A). While the higher molecular weight band matched the size of recombinant murine and human CCL21 in western blot (Fig. 3 A), the lower molecular weight band was similar in size to that of recombinant human CCL21-ΔC (Fig. 3 B). The band ratio did not differ significantly between CTR and CHS-inflamed skin (Fig. 3 C). Moreover, ELISA with an antibody (AF457) recognizing both full-length and cleaved CCL21 (Bastow et al., 2021) revealed similar total CCL21 protein levels in both conditions (Fig. 3 D). In line with the notion that CCL21-ΔC is more soluble than full-length CCL21 (Bastow et al., 2021; Schumann et al., 2010), the lower CCL21 band could be eluted from the ear skin after 24 h of incubation in serum-free medium (Fig. 3 E). Western blot–based quantification of the band intensity indicated an increase of the truncated CCL21 proteoform upon tissue inflammation (Fig. 3, E and F). This finding was confirmed by ELISA performed on the elution medium (Fig. 3 G).

Whereas CCL21-ΔC retains its chemotactic activity (Hauser et al., 2016; Lorenz et al., 2016; Schumann et al., 2010), N-terminal truncation of CCL21 in the range of 32 amino acids reportedly diminishes CCL21-CCR7 binding (Love et al., 2012). In chemotaxis assays (Fig. 3, H–J), we observed that WT, but not CCR7^−/−^, DCs migrated toward the supernatant of the ear skin elution assay, which could be abrogated by antibody-mediated blockade of CCL21 (Fig. 3, H–J). Although the migratory response toward the elution media was rather weak, a consistently higher ratio of WT:CCR7^−/−^ DC migration was observed toward elution media from CHS-inflamed as compared with CTR ear skin (Fig. 3 K). Taken together, these data confirmed that the cleaved CCL21 proteoform represented CCL21-ΔC and that its levels were increased in CHS-inflamed skin, i.e., under conditions in which *in vivo* DC migration was enhanced.

### Two CCL21 proteoforms are present in human skin

To assess the potential relevance of CCL21 cleavage in the human setting, we performed western blot analysis of tissue protein extracts from punch biopsies of healthy human skin. These findings demonstrated that also in human skin two variants of CCL21 exist in a steady state as well as in inflamed skin obtained from psoriasis patients (Fig. 3, L and M).

### LECs express the plasminogen activator uPA and induce CCL21 cleavage *in vitro*

**Figure S1.**
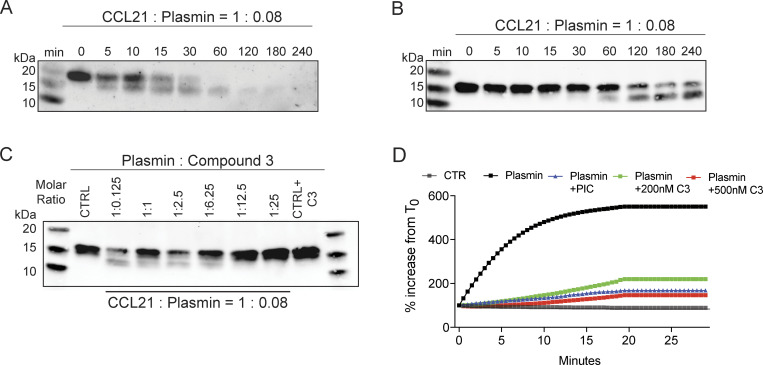
***In vitro* assays demonstrating CCL21 cleavage by plasmin. (A and B)** Western blot analysis of **(A)** human and **(B)** murine CCL21 cleavage after incubation with recombinant plasmin with a fixed molar ratio of 1:0.08 for increasing times at 37°C, as indicated in the figure. **(C)** Dose titration of the plasmin inhibitor C3 to a fixed molar ratio of murine CCL21:plasmin (1:0.08) and incubation for up to 4 h, as indicated in the figure. Representative western blots of *n* = 2 (A) or *n* = 3 (B and C) experiments are shown. **(D)** Fluorometric plasmin activation assay, with indicated plasmin (12 μM) and inhibitor concentrations. The % increase from T_0_ (0 min) is depicted. PIC: broad spectrum protease inhibitor. Representative results of *n* = 3 independent experiments are shown. Source data are available for this figure: SourceData FS1.

**Figure 4.**
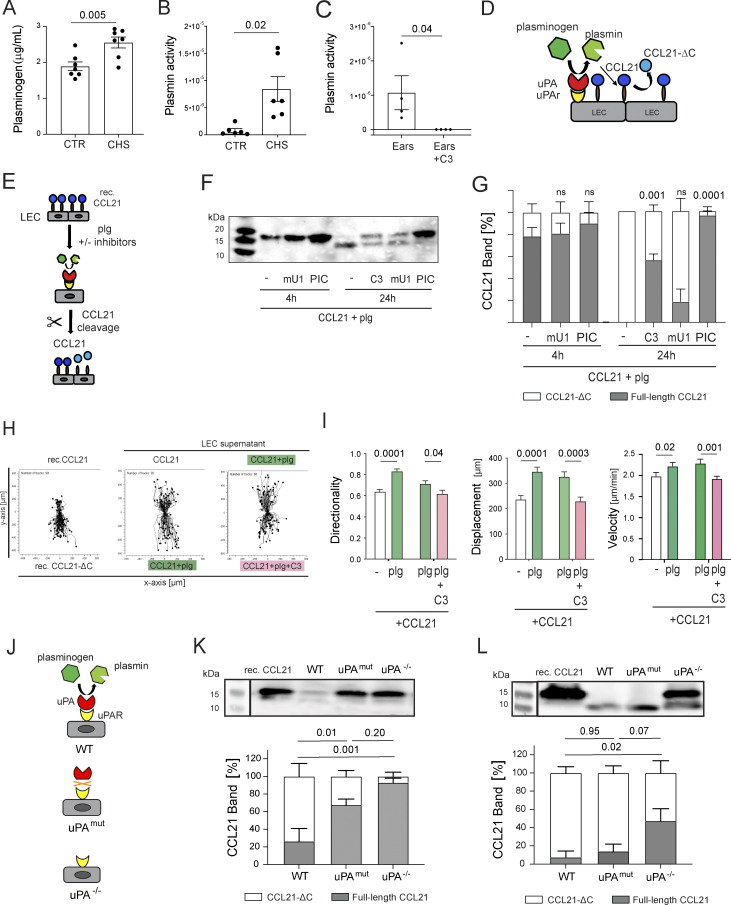
**LECs activate plasminogen to plasmin, thereby generating CCL21-ΔC with enhanced chemotactic activity. (A and B)** Quantification of **(A)** plasminogen and **(B)** plasmin activity in tissue protein extracts generated from CTR or CHS-inflamed ear skin. *n* = 6–7 mice per condition. **(C)** CTR experiment with CHS-inflamed ears documenting that the plasmin activity observed in C can be completely blocked in presence of the plasmin inhibitor C3. **(D)** Schematic depiction of the experimental hypothesis: Inflammation leads to enhanced extravasation of plasminogen. uPA bound to uPAR on CCL21-secreting LECs converts plasminogen to plasmin, thereby inducing CCL21 cleavage into CCL21-ΔC. **(E–G)***In vitro* CCL21 cleavage experiment: **(E)** Schematic depiction of the experiment: immortalized LECs were incubated with recombinant CCL21 (100 nM) and plasminogen (20 nM) for 4 h or 24 h at 37°C in absence or presence of the plasmin inhibitor C3, mU1, or PIC. Supernatants were analyzed by western blot for CCL21. **(F)** Representative western blot of the cell culture supernatant at indicated time points and conditions and **(G)** quantification of the full-length CCL21 (gray) and CCL21-ΔC (white) relative band percentage. Pooled data from *n* = 4 independent experiments. Mean ± SEM, one-way ANOVA, and P values are relative to the “plg only” condition. **(H and I)** Cell culture supernatants generated as in E were evaluated in a 3D collagen migration assay. Recombinant human CCL21 and CCL21-ΔC were used as positive CTRs **(H)** Cell trajectory plots of migrating BMDCs’ migratory tracks in response to the stimuli applied on either side of the collagen channel. **(I)** Quantification of DC directionality, displacement, and velocity in response to the stimuli applied. Pooled data from *n* = 2 independent experiments with a total of *n* = 40–50 tracks analyzed per condition. Mean ± SEM, unpaired Student's *t* test for each comparison. **(J–L)** Analysis of the CCL21 cleavage activity of LECs isolated from uPA^−/−^ mice or mice with defective uPA binding to uPAR (uPA^mut^) **(J)** Schematic illustration of the three genotypes investigated. **(K and L)** Representative western blot of the cell culture supernatants after **(K)** 4 h and **(L)** 24 h of incubation (top) and quantification of the full-length CCL21 (gray) and CCL21-ΔC (white) relative band percentage (bottom). Pooled data from *n* = 5 independent experiments. Mean ± SEM, one-way ANOVA, and Source data are available for this figure: SourceData F4. plg, plasminogen.

The fact that the decrease in perilymphatic CCL21 staining in CHS-inflamed skin was most pronounced immediately at the level of the lymphatics (Fig. 2, C–E) suggested that LECs might not only be the sites of CCL21 production but also of CCL21 cleavage. Since plasmin has previously been shown to cleave human CCL21 *in vitro* (Loef et al., 2022; Lorenz et al., 2016), we set out to investigate whether this protease might contribute to the generation of CCL21-ΔC in the skin. *In vitro* assays performed with human or murine recombinant plasmin confirmed plasmin’s ability to cleave human and murine recombinant CCL21 (Fig. S1, A and B). The reaction could be inhibited by the addition of the broad-spectrum serine protease inhibitor cocktail (PIC) or of the plasmin-selective cyclic peptide inhibitor “Compound 3” (C3) (Swedberg et al., 2019) (Fig. S1, C and D). By ELISA, we detected increased levels of plasminogen in protein extracts generated from CHS-inflamed skin as compared with CTR ear skin of PBS-perfused mice (Fig. 4 A). Moreover, a colorimetric assay showed that plasmin activity was increased in inflamed as compared with CTR skin protein extracts (Fig. 4, B and C). Overall, this suggested that in inflammation, more plasminogen extravasated into the tissue and was converted to plasmin *in situ*, possibly leading to plasmin-induced CCL21 cleavage.

**Figure S2.**
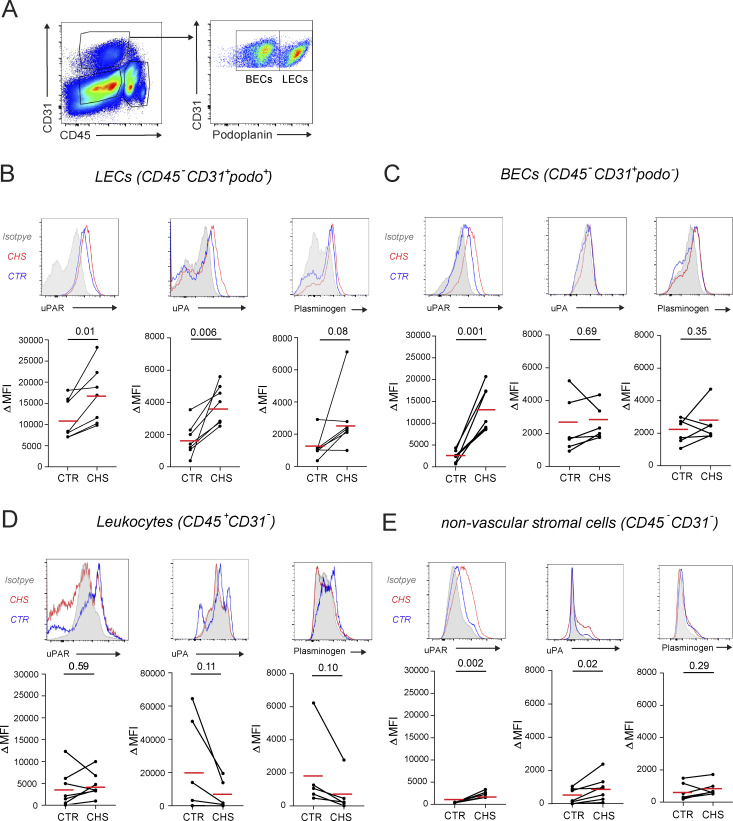
**Flow cytometry–based analysis of uPAR, uPA, and plasminogen protein levels on dermal cell subsets *in vivo*.** Mice were sensitized with 2% oxazolone on the belly on day 0 and challenged on day 5 by applying 1% oxazolone to the skin of one ear. Flow cytometry was performed on both ears, i.e., the CTR and the CHS-inflamed ear, 1 day later. **(A)** Depiction of the gating strategy used for the identification of LECs (CD45^−^CD31^+^podoplanin^+^), blood endothelial cells (CD45^−^CD31^+^podoplanin^-^), leukocytes (CD45^+^CD31^−^), and other nonvascular stromal cells (CD45^−^CD31^−^). **(B–E)** Representative FACS plots (top) and summary of the delta mean fluorescent intensity (ΔMFI; specific*-*isotype staining) values obtained (bottom) when analyzing the expression of uPAR, uPA, and plasminogen in **(B)** LECs, **(C)** blood endothelial cells (BECs), **(D)** leukocytes, and **(E)** other nonvascular stromal cells of CTR or CHS-inflamed skin. Data points from the same animal (i.e., with one CTR and one CHS-inflamed ear, *n* = 4–7 mice in total) are connected by a line. Red lines indicate the mean. Paired Student’s *t* test.

**Figure S3.**
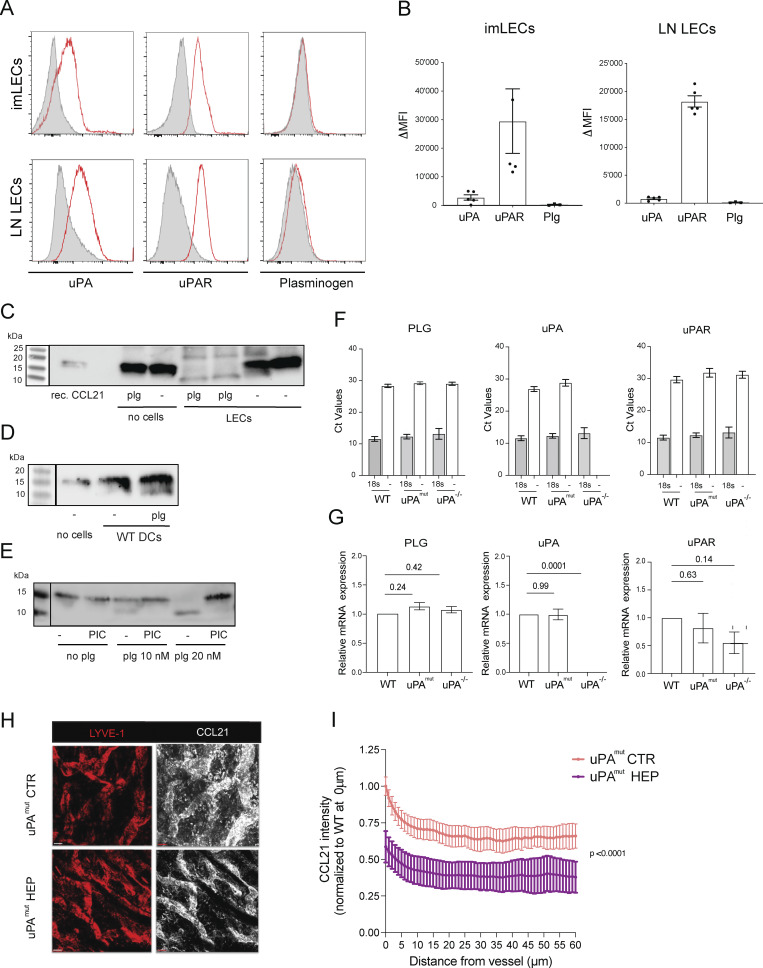
**Expression of components of the plasmin activation pathway and CCL21 cleavage activity of cultured cells. (A and B)** Flow cytometry–based analysis of uPA, uPAR, and plasminogen expression in conditionally immortalized LECs and primary LN LECs. **(A)** Representative histogram plots and corresponding **(B)** summary of the delta mean fluorescent intensity (ΔMFI; specific-isotype staining) values measured in 4–5 different experiments. **(C)** CCL21 cleavage assay performed in presence or absence of LECs and plasminogen (plg), revealing the dependence of CCL21 cleavage on both factors (i.e., LECs and plg). One representative out of two similar experiments is shown. **(D and E)** CCL21 cleavage assay performed with **(D)** bone marrow–derived DCs and **(E)** primary keratinocytes, revealing their ability to cleave CCL21 in presence of plg. One representative out of two similar experiments is shown in D and E. **(F)** qRT-PCR–based analysis of mRNA from LN LECs isolated from WT, uPA^mut^, and uPA^−/−^ mice. **(G)** Absolute CT values and (H) relative expression levels. Data from four LN LEC isolations are shown. One-way ANOVA. **(H and I)** Impact of heparitinase treatment on the CCL21 gradient in uPA^mut^ mice. **(H)** Representative images showing LYVE-1 and the immobilized perilymphatic CCL21 gradient in the steady-state ear skin of uPA^mut^ mice upon *in vitro* treatment with heparitinase (HEP) or in untreated CTRs. Scale bar: 50 μm. (I) Quantification of the CCL21 staining intensity as a function of the distance from the nearest LYVE-1^+^ LV. *n* = 3 mice per condition, two-way ANOVA. Source data are available for this figure: SourceData FS3.

Plasmin is generated through activation of its zymogen plasminogen by either tPA or uPA. Plasminogen-cleavage by tPA is strongly enhanced upon tPA binding to fibrin (Urano et al., 2018), whereas plasminogen cleavage by uPA is fibrin independent but strongly enhanced upon uPA binding to its cell surface–expressed receptor uPAR (Connolly et al., 2010; Ellis et al., 1991). Flow cytometric analysis confirmed presence of uPA, uPAR, and plasminogen on the surface of murine dermal LECs and other dermal cell types and revealed that their combined expression/presence was most consistently upregulated on LECs during CHS-induced inflammation (Fig. S2). Considering that LECs are also the source of CCL21 in the skin, we hypothesized that uPAR- and uPA-expressing LECs might be particularly relevant for uPA-mediated activation of extravasated plasminogen to plasmin, which in turn leads to CCL21 cleavage (Fig. 4 D). To test this hypothesis, we established an *in vitro* assay with immortalized LECs (Vigl et al., 2011), in which the cleavage of CCL21 was investigated in dependency of LEC-induced plasmin activation (Fig. 4 E). Like LECs in the skin, cultured immortalized LECs expressed uPA and uPAR (Fig. S3, A and B). Assays were performed with LEC monolayers cultured for 24 h in the presence of recombinant CCL21 and plasminogen (Fig. 4 E). Western blot analysis after 24 h revealed a conversion of CCL21 to CCL21-ΔC (Fig. 4, F and G). Notably, CCL21 cleavage did not occur when LECs were cultured in absence of plasminogen or upon incubating plasminogen with CCL21 in absence of LECs (Fig. S3 C). Addition of the broad-spectrum serine PIC completely abrogated CCL21 cleavage (Fig. 4, F and G), whereas addition of the plasmin-selective inhibitor C3 (Swedberg et al., 2019) or of the uPA-blocking antibody murine urokinase-neutralizing antibody (mU1) (Lund et al., 2008) resulted in delayed cleavage (Fig. 4, F and G). Overall, we concluded that LECs can activate plasmin, thereby inducing C-terminal cleavage of CCL21. Notably, the ability to cleave CCL21 was not restricted to LECs, as also bone marrow–derived DCs (Schumann et al., 2010) and keratinocytes avidly initiated cleavage of CCL21 upon addition of plasminogen (Fig. S3, D and E).

### LEC-derived CCL21-ΔC elicits stronger chemotactic responses in DCs than full-length CCL21

Since CCL21-ΔC was shown to induce stronger chemotactic responses in DCs than full-length CCL21 (Lorenz et al., 2016; Schumann et al., 2010), we next investigated the chemotactic activity of the CCL21 cleavage product generated by *in vitro*–cultured LECs (Fig. 4 E) in a 3D collagen migration assay (Purvanov et al., 2018). For this, LPS-matured bone marrow–derived DCs were fluorescently labelled and embedded in a collagen matrix between chemokine-containing media reservoirs on each side of the collagen-filled channel, allowing cells to migrate in response to the dominant attractant (Video 3). In agreement with previous reports (Lorenz et al., 2016; Schumann et al., 2010), DCs displayed higher chemotactic activity toward recombinant CCL21-ΔC than toward full-length CCL21 (Fig. 4 H). In the same setup, the supernatant of LECs incubated with “CCL21 + plasminogen” triggered more vigorous DC migration, migratory directionality, and velocity as compared with supernatant of LECs incubated with “CCL21” only, i.e., in absence of plasminogen (Fig. 4, H and I). Addition of C3 abrogated the enhanced migration toward CCL21 + plasminogen supernatant (Fig. 4, H and I). Overall, this indicated that LECs can activate extravasated plasminogen to plasmin, which in turn cleaves CCL21 into the variant CCL21-ΔC, which elicits stronger chemotactic responses (Fig. 4 D).

**Video 3.** **Video showing fluorescently labeled bone marrow–derived DCs (green) moving in 3D collagen toward recombinant human CCL21 (up) and CCL21+plg (down) provided in reservoirs on left and right, respectively, of the chamber.** WT DCs display enhanced migration toward CCL21-ΔC provided on the left. Video specifications: 5-min intervals; 5 frames/s (1500-fold accelerated). The original length of the recording: 200 min. Video length: 8 s. plg, plasminogen.

**Figure S4.**
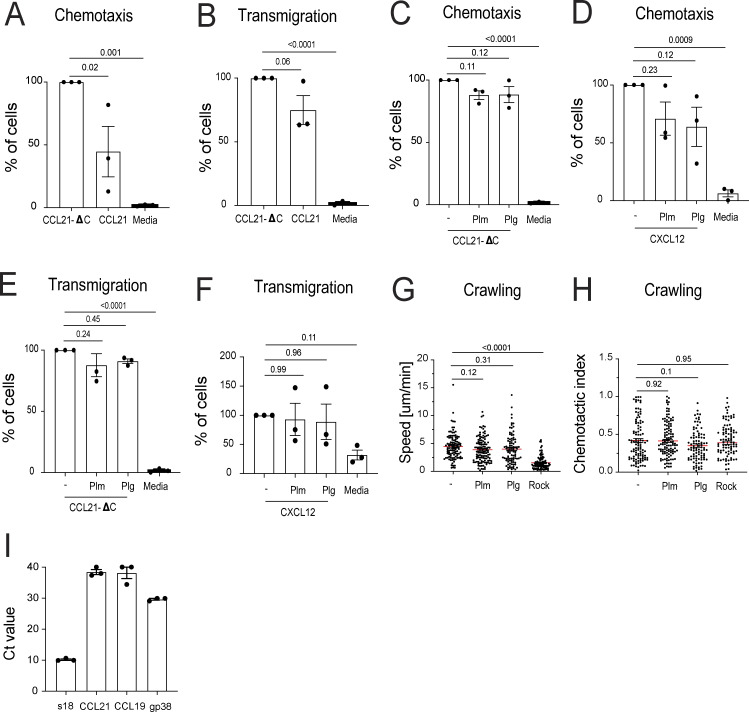
**Impact of plasmin and plasmin(ogen) on *in vitro* DC migration.** *In vitro* experiments were performed with LPS-matured bone marrow–derived DCs and primary LN LECs. **(A)** DC displayed greater chemotaxis toward CCL21-ΔC as compared with full-length CCL21. **(B)** DCs were allowed to transmigrate for 4 h across primary LN LEC monolayers. DCs displayed a near-significant in transmigration (i.e., in two out of three experiments) toward CCL21-ΔC compared with CCL21 added to the lower well compartment. **(C and D)** The presence of plasminogen (Plg) or plasmin (Plm), which were added to the upper and lower wells of the Transwell plate, did not impact DC chemotaxis toward **(C)** CCL21-ΔC and **(D)** CXCL12. Each data point represents an independent experiment. **(E and F)** The presence of plasminogen or plasmin in the assay (upper and lower wells) did not impact DC transmigration across LEC monolayers toward **(E)** CCL21-ΔC or **(F)** CXCL12. Each data point represents an independent experiment. **(G and H)** Crawling assay: YFP-expressing DCs were added on top of LEC monolayers, and their migration was recorded by time-lapse microscopy. The presence of plasminogen or plasmin in the medium did not impact the **(G)** velocity and **(H)** chemotactic index of DC crawling. Of note: Inhibition of ROCK with Y27632 was performed as positive CTR (Nitschke et al., 2012). Statistics: two-way ANOVA using multiple comparisons, followed by a Tukey correction. **(I)** qPCR-results demonstrating that *in vitro*–cultured primary LN LECs no longer express CCL21 and also do not express CCL19. S18: housekeeping gene; gp38: podoplanin. Raw CT values are shown. *n* = 3 different biological replicates (isolations).

Plasmin exerts a variety of effects that could potentially influence DC migration independently of its role in CCL21 cleavage, as previously suggested (Ferrero et al., 2000; Li et al., 2010; Borg et al., 2015). To directly test this, we performed further *in vitro* assays examining the impact of plasmin on the migration of LPS-matured, bone marrow–derived DCs under conditions in which CCL21 cleavage was not relevant. Specifically, we assessed how plasminogen or plasmin affected DC chemotaxis and transmigration across LEC monolayers toward either CCL21-ΔC or CXCL12 presented in the bottom chamber of the Transwell plates. As expected, CCL21-ΔC induced more efficient chemotaxis and slightly enhanced transmigration compared with full-length CCL21 in these assays (Fig. S4, A and B). However, neither plasminogen nor plasmin significantly influenced DC chemotaxis (Fig. S4, C and D) or transmigration (Fig. S4, E and F) toward CCL21-ΔC or CXCL12. Similarly, the presence of plasminogen or plasmin did not affect active DC crawling on LEC monolayers, as determined by time-lapse imaging (Fig. S4, G and H; and Video 4). Importantly, the primary LECs used in these assays expressed virtually no residual endogenous CCL21, as this chemokine is rapidly downregulated in culture (Fig. S4 I). Taken together, these results indicated that *in vitro*, plasmin does not affect DC migration in a CCL21 cleavage-independent manner.

**Video 4.** **Video showing DCs crawling on top of WT LN LEC monolayers.** DCs (green) can be observed crawling on top of LN LECs (gray). Video specifications: 20× objective, 30-s intervals, and 10 frames/s (300-fold accelerated). The original length of the recording: 30 min. Scale bar, 50 μm. Video length: 5 s.

### LECs with lost or diminished uPA activity display reduced CCL21 cleavage

To further investigate the potential involvement of the uPA/uPAR pathway in LEC-mediated plasminogen activation and CCL21 cleavage, we next isolated primary LECs from LNs of either uPA knockout mice (uPA^−/−^) (Carmeliet et al., 1994) or mice expressing a mutated uPA with a 400-fold decreased affinity for murine uPAR (uPA mutant [uPA^mut^]) (Connolly et al., 2010) (Fig. 4 J and further characterization in Fig. S3, F and G). In uPA^mut^ mice, uPA-mediated cleavage of plasminogen to plasmin is severely compromised, whereas other biologic functions of uPA in tissue homeostasis and repair that occur independently of uPAR binding are normal (Connolly et al., 2010). In line with our experiments performed with immortalized LECs (Fig. 4 E), WT, uPA^−/−^, and uPA^mut^ LN LECs were cultured in presence of recombinant CCL21 and plasminogen. Western blot analysis of culture supernatants revealed significantly reduced CCL21 cleavage at 4 h in assays using uPA^mut^ or uPA^−/−^ LN LECs compared with WT LN LECs (Fig. 4 K). At 24 h, the vast majority of CCL21 was cleaved in presence of WT and uPA^mut^ LN LECs, whereas cleavage remained significantly reduced in assays with uPA^−/−^ LN LECs (Fig. 4 L), confirming the contribution of LEC-expressed uPA to CCL21 cleavage (Fig. 4 D).

### Immobilized, perilymphatic CCL21 is also diminished, and CCL21-ΔC increased in 12-O-tetradecanoylphorbol-13-acetate–inflamed skin

**Figure 5.**
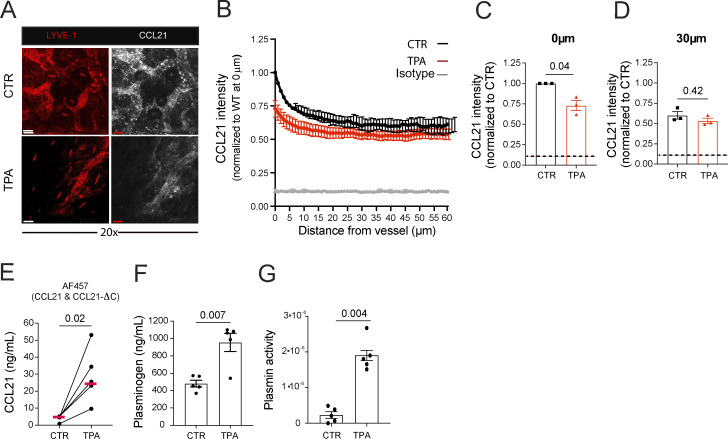
**The immobilized perilymphatic CCL21 gradient is diminished during TPA-induced skin inflammation.** The skin of one ear of each mouse was inflamed by topical application of the irritant TPA. Experiments with both ears, i.e., the uninflamed (CTR and the TPA-inflamed ear, were performed 1 day later. **(A–D)** Analysis of the extracellular, immobilized perilymphatic CCL21 gradient stained in fresh (unfixed) CTR and TPA-inflamed ear skin. **(A)** Representative images showing the CCL21 and LYVE-1 signal at 20× magnification (scale bar: 50 μm). **(B)** Quantification of the CCL21 staining intensity as a function of the distance from the nearest LYVE-1^+^ LV. CCL21 staining intensity was measured at **(C)** 0 μm or **(D)** 30 μm from the LV. *n* = 3 mice per condition (4–6 images analyzed per mouse). The dotted horizontal line in C and D indicates the level of background (isotype) staining. **(E)** Elution assay: CTR and TPA-inflamed ear skin were placed in medium overnight, and the amount of CCL21 protein eluted into the medium was determined by ELISA. **(F and G)** Quantification of **(F)** plasminogen and **(G)** plasmin activity in tissue protein extracts generated from CTR or TPA-inflamed ear skin. *n* = 6–7 mice per condition. All graphs: unpaired Student’s *t* test.

Similar findings as in the CHS model (Fig. 2) suggesting the contribution of LECs to the plasmin-mediated conversion of CCL21-ΔC were also made in an alternative murine inflammation model, namely, upon inducing skin inflammation by topical application of the irritant 12-O-tetradecanoylphorbol-13-acetate (TPA), a potent protein kinase C activator (Friess et al., 2022; Nakadate, 1989). Also in this inflammatory model, the levels of immobilized, perilymphatic CCL21 were most profoundly reduced in the immediate vicinity of LVs (Fig. 5, A–D). At the same time, the amount of CCL21-ΔC recovered from the medium after a 24-h elution assay was significantly increased (Fig. 5 E), indicating enhanced conversion of CCL21 to CCL21-ΔC. Furthermore, as in the CHS model (Fig. 4, A and B), TPA-induced inflammation resulted in elevated tissue plasminogen levels (Fig. 5 F), likely reflecting increased vascular permeability, and was accompanied by enhanced plasmin activity (Fig. 5 G).

### Blockade of uPA activity enhances perilymphatic CCL21 levels while reducing dermal CCL21-ΔC levels

**Figure 6.**
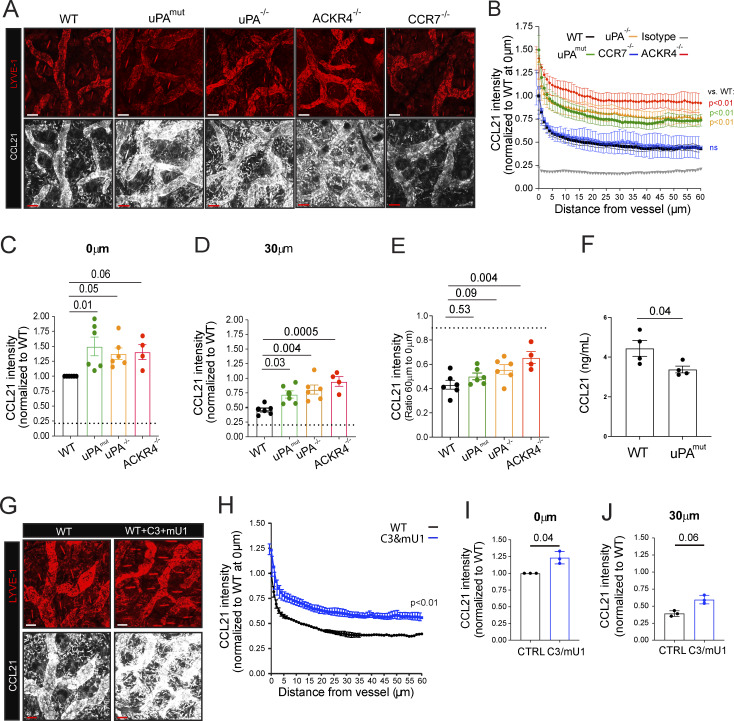
**Blockade of uPA or plasmin activity alters the perilymphatic CCL21 gradient. (A)** Representative images showing LYVE-1 and the immobilized perilymphatic CCL21 gradient in the steady-state ear skin of WT, uPA^mut^ and uPA^−/−^, ACKR4^−/−^, and CCR7^−/−^ mice. Scale bar: 50 μm. **(B)** Quantification of the CCL21 staining intensity as a function of the distance from the nearest LYVE-1^+^ LV. *n* = 4–6 mice per condition, two-way ANOVA **(C and D)** Quantification of the CCL21 staining intensity at **(C)** 0 μm or 30 μm from the LV. **(E)** Ratio of the CCL21 signal intensity measured at 60 vs. 0 μm from the LV. *n* = 4–6 mice per condition; one-way ANOVA. **(F)** ELISA-based quantification of total CCL21 in culture supernatants from ear skin of WT and uPA^mut ^mice, performed with antibody clone AF457 which detects full-length CCL21 and CCL21-ΔC. Pooled data from n = 4 mice/condition. Statistics: unpaired Student's *t* test. **(G–J)** Impact of 24 h of combined treatment with the plasmin-selective inhibitor C3 and uPA-blocking antibody mU1 on the perilymphatic CCL21 gradient. **(G)** Representative images showing the CCL21 and LYVE-1 signal in the ear skin of mice. Scale bar: 50 μm. **(H)** Quantification of the CCL21 staining intensity as a function of the distance from the nearest LYVE-1^+^ LV. Two-way ANOVA, *n* = 3 mice. **(I and J)** Quantification of the CCL21 staining intensity at **(I)** 0 μm or **(J)** 30 μm from the LV. *n* = 3 mice per condition. Paired Student’s *t* test.

To investigate whether loss of uPA or of its ability to bind to uPAR would affect the immobilized perilymphatic CCL21 gradient, we performed whole-mount immunofluorescence in the steady-state ear skin of uPA^−/−^ and uPA^mut^ mice. Notably, in the skin of CCR7^−/−^ mice, in which DC migration is profoundly reduced (Förster et al., 1999), the CCL21 gradient was identical to that observed for WT mice, indicating no contribution of migratory leukocytes to CCL21 cleavage in steady state (Fig. 6, A and B). In line with the gradient analyses reported by Bastow et al. (2021), the immobilized perilymphatic CCL21 gradient in ACKR4^−/−^ mouse skin was markedly elevated and saturated, consistent with impaired scavenging of dermal extracellular CCL21 in the absence of ACKR4 (Fig. 6, A and B). Gradient alterations were also observed in the ear skin of uPA^−/−^ and uPA^mut^ mice, where CCL21 signals were significantly higher than in WT mice, both immediately at the level of the LV and at 30 μm distance from the LV (Fig. 6, A–D). However, unlike the uniformly elevated and saturated gradient observed in ACKR4^−/−^ skin, the gradients in uPA^−/−^ and uPA^mut^ mice retained a more pronounced decline, resembling the profile seen in WT skin (Fig. 6 B). Specifically, the ratio of the CCL21 signal measured at 60 vs. 0 μm from the vessel was not significantly different between WT and uPA^−/−^ and uPA^mut^ mice but was but significantly higher in ACKR4^−/−^ mice (Fig. 6 E). This is likely explained by the continued activity of ACKR4 in dermal keratinocytes and fibroblasts, which contributes to CCL21 scavenging and gradient formation (Bastow et al., 2021), Notably, treatment of uPA^mut^ ear skin explants with heparitinase lead to a partial reduction of the CCL21 signal (Fig. S3, H and I), suggesting that full-length CCL21 continued to be immobilized via heparan sulfates, as previously described (Weber et al., 2013). Moreover, elution assays revealed significantly lower levels of CCL21-ΔC in culture supernatants from uPA^mut^ compared with WT ear skin, indicating reduced CCL21-ΔC tissue levels (Fig. 6 F). Also, treatment of WT mice for 24 h with the plasmin-selective inhibitor C3 and the uPA-blocking antibody mU1 resulted in a similar elevation of the CCL21 gradient (Fig. 6, G–J), revealing the plasticity of the CCL21 gradients *in vivo*. Thus, manipulation of uPA or of its cell surface localization resulted in accumulation of full-length CCL21 around lymphatics, while reducing dermal CCL21-ΔC levels.

### Reduced entry of DCs into dermal lymphatics in uPA^mut^ and uPA^−/−^ mice

**Figure 7.**
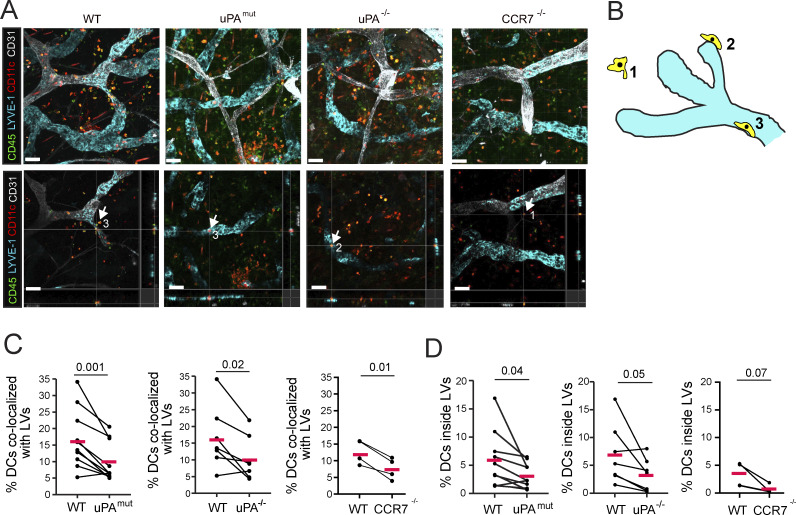
**Loss of uPA or of its cell surface localization alters DC entry into lymphatics.** Quantitative whole-mount analysis of endogenous DC positioning in the steady-state ear skin of WT, uPA^mut^, uPA^−/−^, and CCR7^−/−^ mice. **(A)** Top row: Representative confocal images from the four genotypes. DCs are identified as CD45^+^CD11c^+^ cells (yellow). Bottom row: Confocal images with orthogonal views provided for one selected DC in the upper row image. Scale bars: 50 μm. The number in each image indicates the DC tissue position with respect to the LV, as defined in B. **(B)** Schematic depiction of the three different types of DC tissue positionings: (1) interstitial space, (2) adherent to the outer surface of the LV, and (3) within the LV lumen. **(C)** Quantification of the percentage DCs colocalized with lymphatics (i.e., percentage of (2+3)/(1+2+3), as defined in B). **(D)** Quantification of the percentage DCs localized inside the LV lumen (i.e., percentage of (3)/(1+2+3), as defined in B). Data points from the same experiment (involving one mouse per genotype) are connected by a line. *n* = 4–10 mice per condition. Paired Student’s *t* test.

The literature and our experimental data so far suggest that the presence of soluble CCL21-ΔC, forming a far-reaching gradient into the tissue, may be critical for recruiting DCs from more distant sites toward and into LVs. We therefore sought to investigate whether a shift in the CCL21 balance—from soluble CCL21-ΔC to more immobilized CCL21, as observed in uPA^mut^ mice (Fig. 6, A–F)—would negatively affect DC migration into dermal lymphatics. Since traditional *in vitro* crawl-in assays using bone marrow–derived DCs and ear skin explants (Fig. 1, B and C) capture only the effects of the immobilized, full-length CCL21, and since soluble CCL21-ΔC is not retained in the tissue upon exposing the dermal aspect, we instead examined the positioning of endogenous DCs (CD45^+^CD11c^+^) around lymphatics in intact skin by whole-mount immunofluorescence (Fig. 7 A). Specifically, we performed blinded image analysis to distinguish DCs in the interstitium, adhering to the outside of lymphatics or DCs within the LV lumen (Fig. 7, A and B). Quantification revealed a significantly lower percentage of DCs colocalized with lymphatics in uPA^−/−^ and uPA^mut^ compared with WT ear skin (Fig. 7 C). Similarly, the percentage of DCs found within the LV lumen was decreased in uPA^−/−^ and uPA^mut^ ear skin (Fig. 7 D). Overall, these findings indicated that the soluble and immobilized CCL21 gradients are continuously shaped by uPA-mediated CCL21 cleavage and that soluble CCL21-ΔC levels are critical for DC migration toward and into afferent lymphatics.

### Loss of uPA-mediated CCL21 cleavage reduces CCL21-ΔC levels in dLNs

**Figure 8.**
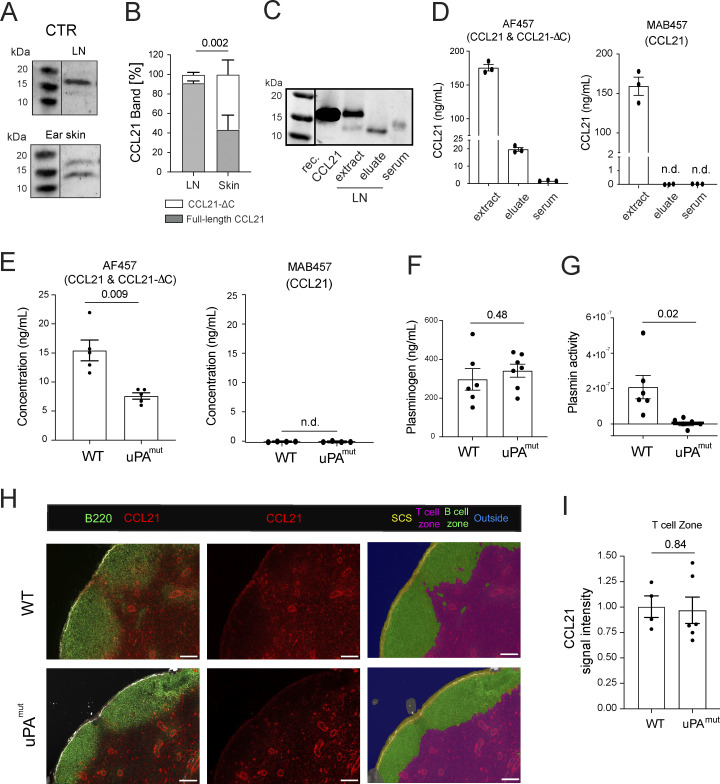
**Blockade of uPA results in reduced CCL21-ΔC levels in skin-dLNs. (A and B)** Western blot–based comparison of CCL21 proteoforms in steady-state LNs and ear skin. **(A)** Representative western blots performed on tissue protein extracts and **(B)** and quantification of the full-length CCL21 (gray) and CCL21-ΔC (white) relative band percentages. Pooled data from *n* = 4 western blots from independent experiments. **(C)** Representative western blot of CCL21 performed on LN extracts, LN eluates, and serum. **(D)** ELISA-based quantification of full-length CCL21 and CCL21-ΔC present in LN extracts, LN eluates, or serum. Antibody clone MAB457 detects full-length CCL21 only, whereas clone AF457 detects both full-length CCL21 and CCL21-ΔC. Data from *n* = 3 mice are shown. n.d., not detected. **(E)** ELISA-based quantification of full-length CCL21 and CCL21-ΔC present in LN eluates from WT and uPA^mut^ mice. Data from *n* = 4–5 mice are shown, Student’s *t* test. n.d., not detected. **(F and G)** Protein extracts were prepared from LNs of PBS-perfused WT and uPA^mut^ mice and used for ELISA-based quantification of (F) plasminogen and (G) assessment of plasmin activity (colorimetric assay). Pooled data from *n* = 6 mice per group are shown as mean ± SEM. Student’s *t* test. **(H and I)** Analysis of CCL21 levels in LNs of WT and uPA^mut^ mice. Freshly cut LN sections were immediately (i.e., without fixation/permeabilization) stained for B220 (B cell follicles), LYVE-1, and CCL21. Images were subjected to AI-based tissue segmentation for differentiating between the T cell zone and B cell follicles/SCS. (H) Representative images from the immunofluorescent staining performed on LNs of WT and uPA^mut^ mice. Scale bar: 100 μm. (I) Quantification of CCL21 staining intensity observed in the T cell zone. Each dot represents data from a stained auricular LN of one mouse (average of 4–6 images per LN). Student’s *t* test. Source data are available for this figure: SourceData F8.

Besides DC chemotaxis and entry into afferent lymphatics, CCL21 gradients also guide intralymphatic crawling of DCs from capillaries toward lymphatic collectors (Russo et al., 2016) as well as DC egress from the SCS and migration into the CCL21-rich T cell zone (Braun et al., 2011; Ulvmar et al., 2014) (Fig. 1 A). Since DC entry into dermal lymphatic capillaries was compromised in uPA^−/−^ and uPA^mut^ mice (Fig. 7), it seemed likely that also the subsequent CCR7-dependent steps, such as egress from the SCS, might be altered upon impairment of CCL21 cleavage. We therefore next investigated CCL21 proteoforms in skin dLNs. In contrast to the skin, CCL21 was primarily present in its full-length form in protein extracts of LNs draining steady-state skin (Fig. 8, A and B). On the other hand, upon placing intact LNs in medium overnight (Fig. 8 C), only CCL21-ΔC eluted into the supernatant, indicating that mainly CCL21-ΔC was present in the SCS and connecting lymphatics. In agreement with this hypothesis, only CCL21-ΔC was detected by western blot in murine serum (Fig. 8 C). These results were also confirmed by performing ELISA with antibodies detecting either only full-length CCL21 or both CCL21 proteoforms (MAB457 and AF457, respectively [Bastow et al., 2021]) (Fig. 8 D). Overnight elution assays performed on intact LNs revealed significantly lower levels of CCL21-ΔC in the culture medium of uPA^mut^ LNs compared with WT (Fig. 8 E). We next investigated plasminogen levels and plasmin activity in LNs of WT and uPA^mut^ mice. Whereas plasminogen levels were similar in LNs from both genotypes (Fig. 8 F), plasmin activity was profoundly decreased in LNs from uPA^mut^ mice (Fig. 8 G), suggesting that uPA/uPAR/plasmin might similarly contribute to CCL21 cleavage in LNs. Surprisingly, when analyzing the immobilized, extracellular CCL21 levels in fresh LN sections prepared from WT and uPA^mut^ mice, no difference was observed in CCL21 intensities (Fig. 8, H and I). Overall, the reduced levels of CCL21-ΔC observed in LN eluates (Fig. 8 E) indicated that in steady state less CCL21-ΔC is present in the SCS in uPA^mut^ LNs, potentially facilitating CCR7-dependent DC exit across the SCS (Ulvmar et al., 2014).

### Loss of uPA-mediated CCL21 cleavage enhances DC egress from the SCS into the LN parenchyma

**Figure S5.**
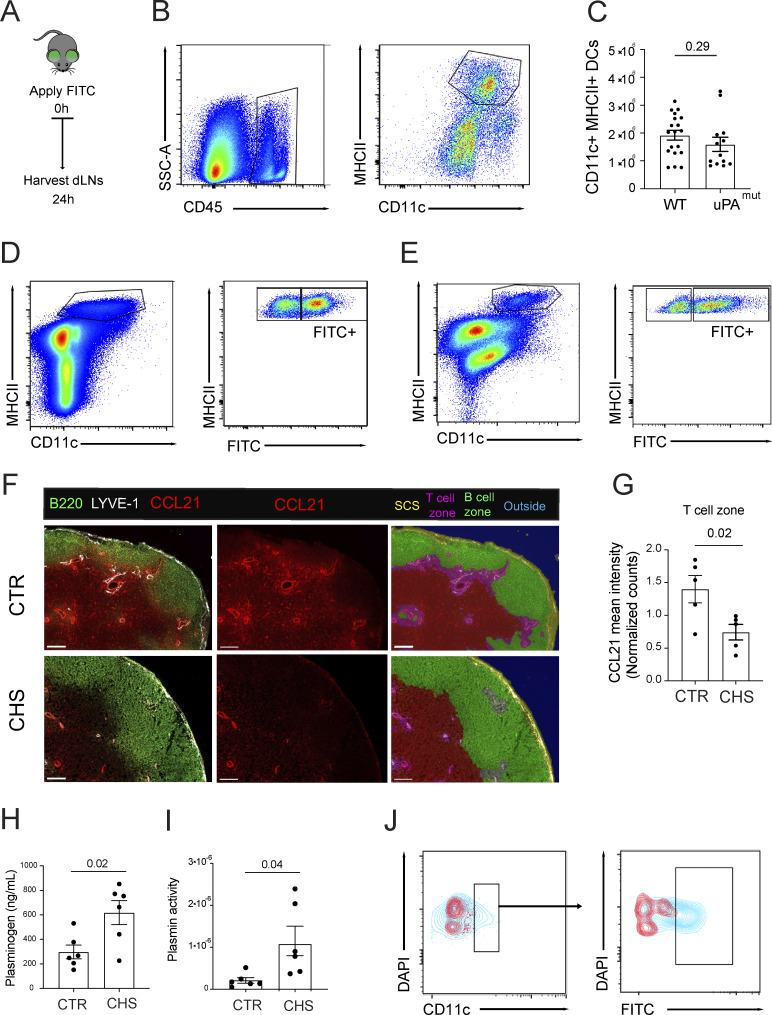
**Supplemental data to FITC painting experiments and analysis of LNs draining CHS-inflamed skin. (A)** Schematic depiction of the experiment: FITC was applied to the ear skin in WT and uPA^mut^ mice, and ear skin or ear-draining auricular LNs were collected for analysis after 24 h. **(B and C)** Analysis of dermal DC numbers. Ear skin was enzymatically digested, and single-cell suspensions were generated for flow cytometry–based analysis. **(D and E)** Gatings used to identify migratory DCs (CD11c^+^ MHCII^hi^), and, amongst those, FITC^+^ DCs in single-cell suspensions generated from (D) enzymatically digested LNs and (E) undigested LNs. **(F–I)** Analysis of immobilized CCL21 and plasmin(ogen) in CHS-dLNs. A CHS response was induced in the ear skin of WT mice, and ear-draining auricular LNs were collected 24 h later. (F and G) Analysis of CCL21 levels in LNs draining CTR or CHS-inflamed ear skin. Freshly cut LN sections were immediately (i.e., without fixation/permeabilization) stained for B220 (B cell follicles), LYVE-1, and CCL21. (F) Representative images of the immunofluorescent staining and of the AI-based tissue segmentation used for differentiating between the T cell zone and B cell follicles/SCS. Scale bar: 100 μm. (G) Quantification of CCL21 staining intensity of observed in the T cell zone. Each dot represents data from a stained auricular LN of one mouse (average of 4–6 images per LN). Student’s *t* test. (H) ELISA-based quantification of (H) plasminogen and (I) plasmin activity in LN protein extracts, generated after perfusing the mice with PBS. Pooled data from *n* = 6 mice per group are shown in H and I, Student’s *t* test. **(J)** Representative gating strategy used for the quantification of FITC^+^CD11c^+^ cells in LN sections from WT and uPA^mut^ FITC-painted auricular LNs in Fig. 9, E–G. CD11c^+^ cells were identified based on CD11c-AF647 positivity. From the CD11c^+^ population, FITC^+^ cells were identified and subsequently quantified. Marker-negative cells (red color) served as negative control to check background staining and to set the fluorescence thresholds. Please note that the data points of the WT CTR group in C are identical to those shown in Fig. 8 F, as these extracts were prepared and measured simultaneously.

**Figure 9.**
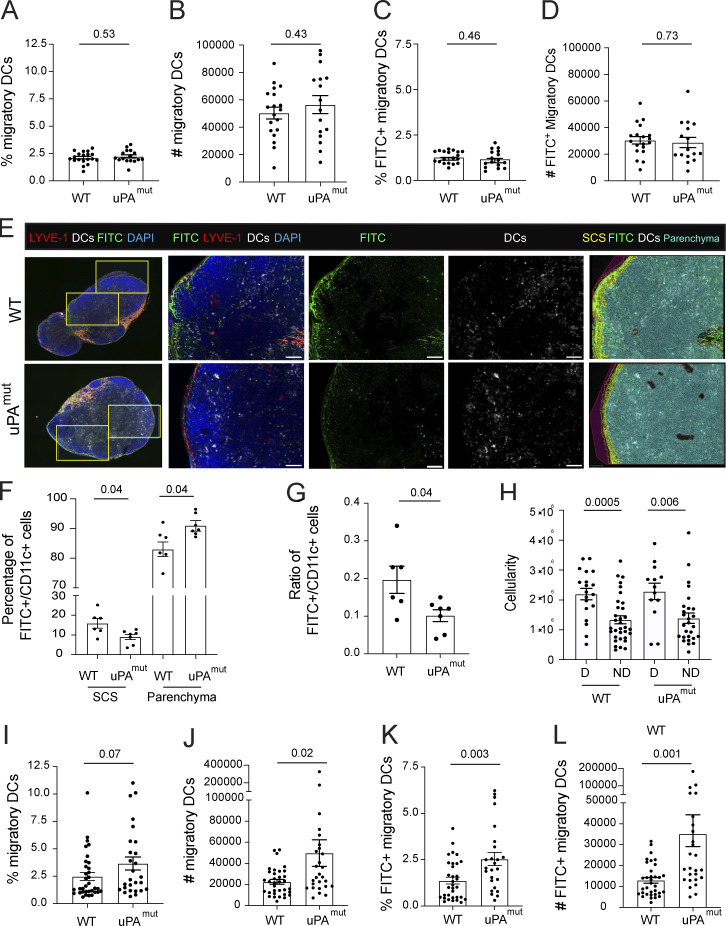
**DC migration from skin into the LN parenchyma is enhanced in uPA** ^**mut**^ **mice.** FITC was applied to the ear skin in WT and uPA^mut^ mice, and ear-draining auricular LNs were collected for analysis after 24 h. **(A–D)** Flow cytometry–based quantification of DCs in single-cell suspensions of enzymatically digested LNs. Percentage (A and C) and absolute numbers (B and D) of all migratory DCs (A and B; CD11c^+^MHCII^+^) and FITC^+^ migratory DCs (C and D). Pooled data from two similar experiments are shown (*n* = 16–19 mice per group). **(E–G)**. Immunofluorescence-based analysis of sections prepared from auricular LNs after FITC painting. **(E)** Representative whole-slide multiplex immunofluorescence images of WT and uPA^mut^ LNs. Images on the far left: overall LN architecture and examples of regions of interest (yellow boxes) used for high-resolution analysis of the SCS and parenchymal compartments. Subsequent images (left to right): higher-magnification images showing a merge of FITC signal, CD11c (DCs) and LYVE-1 (LVs) staining, followed by single-channel views of FITC and CD11c. Images on the far right: AI-based segmentation maps of FITC⁺CD11c⁺ DCs in the SCS or parenchyma. Scale bar.: 100 μm. **(F)** Percentage of FITC^+^CD11c^+^ DCs localized in the SCS or in the LN parenchyma, and **(G)** ratio of FITC^+^CD11c^+^ DCs in the SCS vs. LN parenchyma in WT and uPA^mut^ LNs. Pooled data from the analysis of 6–7 mice per genotype are shown. Each dot presents the average of 3–6 images analyzed per one mouse. **(H)** Comparison of the LN cellularity retrieved from enzymatically digested (“D”) or nondigested (“ND”) LNs. Data points belong to the experiments described in A–D; “D” and I–L, “ND”. **(I–L)** Flow cytometry–based quantification of DCs in single-cell suspensions generated not digested LNs. Percentage and absolute numbers of **(I and J)** all migratory DCs (CD11c^+^MHCII^+^) and **(K and L)** FITC^+^ migratory DCs. Pooled data from three similar experiments are shown (*n* = 31–34 mice per group). Unpaired Student’s *t* test (all graphs).

**Figure 10.**
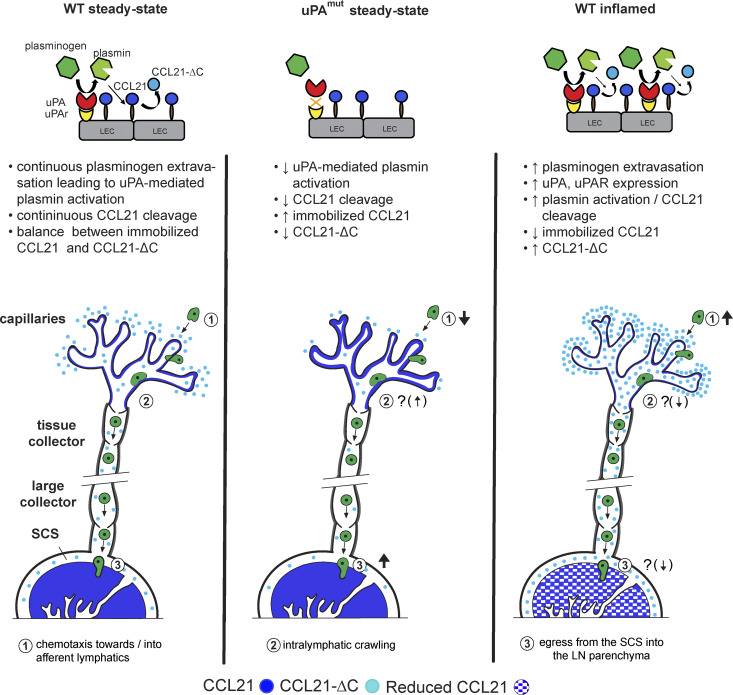
**Summary diagram.** Summary of the main findings and the overall model. Top: Summary of events happening at the level of LECs: continuous low-level extravasation of plasminogen from blood vessels leads to uPA/uPAR-mediated activation of plasmin, which in turn cleaves immobilized CCL21 into soluble CCL21-ΔC (WT steady-state—left). When uPA-mediated activation of plasminogen is compromised (uPA^mut^), less CCL21 gets cleaved, shifting the balance toward more immobilized CCL21 accumulating on/around LECs (uPA^mut^ steady-state—middle). Under inflammatory conditions, with higher extravasation of plasminogen and higher expression of uPA and uPAR by LECs, more plasmin is activated, resulting in more CCL21 cleavage (WT inflammation—right). Bottom: The bottom part of the figure illustrates how these changes affect the balance between immobilized CCL21 and soluble CCL21-ΔC around afferent lymphatics and in the dLN. Additionally, the impact on distinct CCR7-dependent steps (1–3) in lymphatic migration of DCs are indicated. Question marks (?) indicate steps that were not specifically investigated in this study and thus represent speculations based on indirect findings and/or the literature.

To investigate how impairment of plasmin/uPA/uPAR-mediated CCL21 cleavage would affect overall DC migration to dLNs (Fig. 1 A), we performed FITC painting experiments in the ear skin of WT and uPA^mut^ mice. In this assay, the fluorescent dye FITC is applied to the skin and is taken up by dermal DCs, enabling the tracking of their migration to skin-dLNs. 24 h after FITC application, ear skin and ear-draining auricular LNs were harvested and enzymatically digested to quantitatively release DCs from both the LN parenchyma and SCS (Fig. S5 A) (Sokol et al., 2018). Flow cytometry–based quantification revealed no difference in absolute DC numbers in the digested ear skin of WT and uPA^mut^ mice (Fig. S5, B and C). Similarly, the percentage and number of total migratory DCs and of FITC^+^ migratory DCs in LNs were comparable between genotypes (Fig. 9, A–D, gating in Fig. S5 D), suggesting that the defect in DC entry into dermal lymphatics (Fig. 7) had been compensated during subsequent migratory steps (Fig. 1 A). However, immunofluorescence analysis of auricular LNs revealed that a higher proportion of FITC^+^ DCs were retained within the SCS in WT mice compared with uPA^mut^ mice (Fig. 9, E–G). To confirm that DCs in uPA^mut^ mice more efficiently entered the LN parenchyma from the SCS, we conducted additional FITC painting experiments, this time generating LN single-cell suspensions from mechanically dissociated, non-digested LNs. This approach has been shown to preferentially recover DCs from the LN parenchyma, but not from the SCS. Accordingly, comparing results from undigested with those from digested LNs provides a means to detect differences in SCS egress (Sokol et al., 2018). As expected, the total cell yield from non-digested LNs was significantly reduced compared with enzymatically digested samples (Fig. 9 H). In agreement with our microscopy-based analysis (Fig. 9, E–G), both the number and frequency of FITC^+^ DCs recovered from uPA^mut^ LNs were significantly higher than in WT LNs using this method (Fig. 9, I–L, gating in Fig. S5 E). Together, these findings showed that impairment of CCL21 cleavage enhanced DC exit from the SCS into the LN parenchyma, revealing that uPA/uPA/plasmin-mediated CCL21 cleavage regulates yet another CCR7-dependent steps important for DC migration (summarized in Fig. 10).

## Discussion

The chemokine CCL21 and its receptor CCR7 are considered the most important drivers of DC migration (Förster et al., 1999; Ohl et al., 2004; Permanyer et al., 2018). Although the contribution of full-length CCL21 to this process is well established, it was thus far unknown how CCL21-ΔC impacts *in vivo* migration and how this more soluble CCL21 variant, making it more effective at supporting chemotaxis, is generated in tissues. In this study, we demonstrate that uPA/uPAR/plasmin-mediated CCL21 cleavage is responsible for the generation of CCL21-ΔC in murine skin and that the balance between the two proteoforms regulates at least two CCR7-dependent steps in DC migration.

Our experiments suggest that tissue-immobilized and soluble CCL21 gradients act in concert in directing DC migration toward and into dermal lymphatics. The important contribution of CCL21-ΔC to this process explains why migration of adoptively transferred DCs from CHS-inflamed skin to dLNs was enhanced despite a strongly diminished immobilized perilymphatic CCL21 gradient. Conversely, perilymphatic accumulation of immobilized, full-length CCL21 combined with reduced CCL21-ΔC tissue levels in uPA^mut^ mice resulted in reduced DC migration into dermal afferent lymphatics. Together with our *in vitro* 3D-collagen chemotaxis assays, demonstrating efficient attraction of DCs by LEC-generated CCL21-ΔC, these findings support our conclusion that partial conversion of the immobilized full-length CCL21 to CCL21-ΔC supports steady-state DC migration toward lymphatics by forming an additional, far-reaching, and largely soluble gradient. Our findings provide *in vivo* support and expand a previous *in vitro* study showing that immobilized and soluble CCL21 cooperate for optimal DC migration (Schumann et al., 2010).

Tissue fluids are constantly transported from the interstitial space into draining lymphatics (Bazigou et al., 2014; Wiig and Swartz, 2012). Considering this, it might appear puzzling that CCL21-ΔC had any positive impact on DC migration and was able to establish a far-reaching perilymphatic gradient in the opposite direction of the interstitial fluid flow. In the case of full-length CCL21, the establishment of the perilymphatic gradient is thought to depend on CCL21’s strong cell surface and extracellular matrix-binding capacity, which is mainly attributed to its positively charged C terminus (Hjorto et al., 2016; Moore and Bertram, 2018). However, although the stickiness of CCL21-ΔC is strongly diminished, it is not completely abolished (Artinger et al., 2022; Artinger et al., 2023; Hjorto et al., 2016), suggesting that CCL21-ΔC still retains residual, transient tissue-binding activity that supports the formation of a soluble perilymphatic gradient. In line with this hypothesis, we found CCL21-ΔC levels to be significantly enhanced in CHS-inflamed tissues and reduced in uPA^mut^ mice (Fig. 10, A–C), indicating that also the more soluble chemokine is not immediately cleared from the tissue via lymphatic drainage.

Plasmin was previously shown to cleave CCL21 *in vitro* (Lorenz et al., 2016), and tPA on activated T cells has been implicated in this cleavage process (Loef et al., 2022). Here, we found that plasmin indeed cleaved CCL21 *in vivo* and identified a role for the second plasminogen activator, i.e., uPA, in plasmin activation leading to CCL21 cleavage in the skin. Although we detected uPA and uPAR on various cell types in the murine ear skin, we observed that their combined expression was most consistently upregulated in LECs under inflammatory conditions. The fact that LECs are at the same time the source of CCL21 makes them the most obvious major cell type responsible for CCL21 cleavage and likely explains the profound loss of CCL21 staining intensity observed right at the level of the lymphatics in CHS- or TPA-inflamed skin. Conversely, reduction of plasmin-mediated CCL21 cleavage in uPA^mut^ and uPA^−/−^ mice resulted in a pronounced accumulation of immobilized CCL21 around lymphatics (Fig. 10, A–C). Importantly, the same phenotype could be pharmacologically recapitulated by interfering with plasmin activation/activity for 24 h, revealing the dynamic nature of CCL21 cleavage and gradient regulation.

Our skin and LN elution experiments predominantly detected CCL21-ΔC, indicating that this is the principal CCL21 proteoform present in tissue fluids, such as in lymphatics, as well as in the SCS compartment. In line with this observation, it was recently found that skin-derived CCL21-ΔC accumulates in the SCS of the dLN (Bastow et al., 2021). In contrast to skin tissue, which was composed of both full-length CCL21 and CCL21-ΔC, only a minute fraction of CCL21 in steady-state LNs was present as CCL21-ΔC. We therefore suspect that CCL21 cleavage in dLNs occurs at a lower rate compared with the skin. This interpretation is supported by our immunofluorescence analyses, as we did not detect an accumulation of immobilized CCL21 in steady-state LNs of uPA^mut^ mice. Notably, the immobilized CCL21 signal in WT LNs was already very high, though, raising the possibility that the assay approached saturation and might therefore have masked subtle genotype-dependent differences. In contrast, we did observe a reduction in immobilized CCL21 levels in LNs draining CHS-inflamed skin, accompanied by increased plasminogen levels and plasmin activity (Fig.S5, F–I). Interestingly, two previous studies reported that during infection, CCL21 levels are profoundly downregulated in the spleen (Mueller et al., 2007) and in LNs (Chen et al., 2025), both at the protein and at the mRNA level. This indicates that regulation of CCL21 expression and availability in secondary lymphoid organs, where fibroblastic reticular cells are the main CCL21 producers (Li et al., 2021; Link et al., 2007), might be different from that in dermal afferent LVs.

Our experiments in uPA^mut^ mice demonstrate that continuous CCL21 cleavage around dermal lymphatics—and the resulting shift in the balance between immobilized full-length CCL21 and CCL21-ΔC—affects not only DC chemotaxis toward lymphatics but also subsequent migratory steps (summarized in Fig. 10). While DC entry into dermal lymphatic capillaries was reduced, the total number of DCs recovered from digested dLNs was comparable between uPA^mut^ and WT mice, suggesting that diminished CCL21 cleavage may have facilitated intralymphatic DC crawling sufficiently to compensate for reduced vessel entry. This effect could result from the lower abundance of CCL21-ΔC in lymphatic capillaries of uPA^mut^ mice, thereby improving haptotactic DC crawling along the flow-deposited immobilized CCL21 gradient (Russo et al., 2016), enabling DCs to reach collecting vessels more rapidly and be transported to the SCS with greater efficiency (Fig. 10, A and B). In the SCS, lower levels of skin-derived CCL21-ΔC in uPA^mut^ mice likely facilitate DC egress into the LN parenchyma by reducing soluble chemokine levels that would otherwise obscure the detection of parenchymal immobilized CCL21. This interpretation is further supported by findings from a recent study showing that in ACKR4^−/−^ mice, excess CCL21-ΔC from upstream tissues impairs DC emigration from the SCS into the LN parenchyma (Bastow et al., 2021).

Although our data identify plasmin as a regulator of *in vivo* DC migration through its ability to shift the balance between full-length, immobilized CCL21 and cleaved, soluble CCL21, it is important to note that plasmin exerts additional effects relevant to tissue dynamics. Beyond CCL21 processing, plasmin can degrade extracellular matrix proteins, activate latent matrix metalloproteinases, and modulate the activity of growth factors and cytokines, all of which could directly or indirectly influence DC migration within tissues (Heissig et al., 2020; Keragala and Medcalf, 2021). Our *in vitro* assays, examining DC crawling on LEC monolayers as well as DC chemotaxis and trans-endothelial migration toward CXCL21 or CCL21-ΔC, did not reveal further plasmin- or plasminogen-dependent effects on DC motility. Nevertheless, given the broad impact of plasmin on tissue homeostasis, we cannot exclude the possibility that additional plasmin-dependent pathways contribute to DC migration in the more complex *in vivo* environment.

Overall, our data reveal the highly dynamic nature of immobilized and soluble CCL21 gradients and how shifting their balance fine-tunes DC migration. But what could be the immunological benefit of such continuous gradient adjustments? Based on our findings, we suspect that under inflammatory conditions, where DC migration is enhanced, plasmin-mediated CCL21 cleavage rapidly shifts the balance toward more CCL21-ΔC, resulting in enhanced DC migration into lymphatics (Fig. 10). Conversely, once within capillaries, the presence of high levels of CCL21-ΔC would be expected to confound intralymphatic DC migration by obscuring haptotactic migration along the flow-deposited CCL21 gradient. Likewise, similarly as in ACKR4^−/−^ mice (Bastow et al., 2021; Ulvmar et al., 2014), DC exit from the SCS into the LN node should be compromised due to increased CCL21-ΔC levels in the SCS combined with reduced levels of immobilized CCL21 in the LN parenchyma. Considering that we found *in vivo* DC migration from skin to dLNs to be even more CCR7 dependent in CHS than under steady-state conditions (Fig. 1 and [Vigl et al., 2011]), a shift in the overall balance from full-length CCL21 to CCL21-ΔC during inflammation could serve to ensure in infection-induced settings that preferentially fully activated DCs—which would be expected to be CCR7^hi^ following direct pathogen-driven maturation and to present foreign antigen—reach the dLNs. In contrast, bystander-activated DCs, which might preferentially present self-antigens and express lower CCR7 levels due to a lack of direct contact with pathogenic constituents, would be expected to be at a migratory disadvantage. Although purely speculative at this point, this type of CCL21 gradient regulation could contribute to the maintenance of tolerance.

## Materials and methods

### Mice

WT C57BL/6J mice were purchased from Janvier. CCR7^−/−^ (Förster et al., 1999), CD11c-YFP (Lindquist et al., 2004), Prox-1-mOrange (Hägerling et al., 2011), uPA^mut^ (Connolly et al., 2010), and uPA^−/−^ (Carmeliet et al., 1994) mice were bred in our facility. Prox-1-mOrange × CD11c-YFP were generated by intercrossing the parental mouse strains. All mice were housed in specific pathogen-free conditions at the facilities RCHCI and EPIC of ETH Zurich. Male and female mice ranging from 6–12 wk were used for experiments. All experiments were approved by the Cantonal Veterinary Office Zurich and performed under experimental licenses ZH 237/16, ZH 239/19, and ZH 053/2023.

### Antibodies

The clone, catalogue number, concentration, and manufacturer of all primary and secondary antibodies used for flow cytometry, immunostaining, western blot, and ELISA are provided in Table S1.

### Primary cells and cell lines

#### Generation of bone marrow–derived DCs

Bone marrow–derived DCs were generated and cultured as previously described (Lutz et al., 1999). In brief, BM was isolated from femurs and tibia of WT or CCR7^−/−^ mice and cultured for 6–9 days on bacterial dishes in DC medium (Roswell Park Memorial Institute Medium [RPMI] 1640, 10% FBS, 15 nM HEPES, 1 mM sodium pyruvate, penicillin (100 U/ml), streptomycin (100 μg/ml), 2 nM L-glutamine, and 50 μM β-mercaptoethanol; purchased either from Gibco or from Sigma-Aldrich) in a humidified incubator at 37°C and 5% CO_2_. The medium was further supplemented with granulocyte-macrophage colony-stimulating factor (GM-CSF) derived from the supernatant of myeloma cells (X63 Ag8.653) transfected with murine GM-CSF complementary DNA (cDNA) (Zal et al., 1994). The medium was exchanged on day 3 and on day 6. After 7–9 days, cells were transferred to TPP cell culture–treated plates (Thermo Fisher Scientific) to deplete the culture of macrophages, which adhere to the culture plate, and were treated overnight with 200 ng/ml LPS from *Salmonella* abortus equi S-form (Enzo Life Sciences) to induce DC maturation.

#### Isolation of LN LECs

LN LECs were isolated from WT, uPA^mut^, or uPA^−/−^ mice as previously described (Arasa et al., 2021b). In brief, pooled cervical, auricular, branchial, inguinal, and popliteal LNs from 2–4 adult mice were digested with 0.25 mg/ml Liberase DH (Roche) and 200 U/ml DNase I (Sigma-Aldrich) in RPMI medium (Gibco) for 1 h at 37°C while being mechanically disrupted by pipetting every 15 min. LN cell suspensions were filtered and plated in LN LEC medium (MEM alpha medium, 10% FBS, penicillin (100 U/ml), and streptomycin (100 µg/ml), all from Gibco) on TPP cell culture plates coated with collagen (10 μg/ml; Advanced BioMatrix) and fibronectin (10 μg/ml; Millipore). Cells were grown until confluency in a humidified incubator at 37°C and 5% CO_2_, with regular media exchange to remove nonadherent cells. Subsequently, cells were detached with Accutase (Gibco), and endothelial cells were selected by CD31^+^ microbead selection, following the manufacturer’s protocol (Miltenyi Biotec). The identity of LN LECs was confirmed by flow cytometry–based analysis of CD31 and podoplanin expression using the rat-anti-mouse CD31 APC (clone MEC 13.3; BioLegend) and the Syrian hamster anti-mouse Podoplanin PE/Cy7 (clone 8.1.1, BioLegend) antibodies. LECs were cultured and used for *in vitro* assays up to passage four.

#### Immortalized murine LECs

Immortalized LECs expressing a heat-labile version of the large T antigen (Vigl et al., 2011) were cultured in a humidified incubator at 33°C and 5% CO_2_ on TPP cell culture plates coated with collagen (10 μg/ml; Advanced BioMatrix) and fibronectin (10 μg/ml; Millipore) in DMEM/F-12 GlutaMAX media, supplemented with 20% FBS (both from Gibco), 56 μg/ml heparin (Sigma-Aldrich), 10 μg/ml endothelial cell mitogen (AbD Serotec), antibiotic antimycotic solution (1×; FLUKA), and L-glutamine (2 nM; FLUKA). Additionally, murine IFN-γ(1 U/ml, PeproTech) was added to induce large T-antigen expression. For experimental assays, immortalized LECs were seeded on collagen- and fibronectin-coated dishes without IFN-γ and cultured in a humidified incubator at 37°C and 5% CO_2_ for at least 24 h prior to starting the assays.

#### Murine immortalized keratinocytes

Isolated keratinocytes from mouse skin (kind gift of Marco Sesartic [ETH Zurich]) were cultured in keratinocyte media, consisting of three parts: a defined keratinocytes serum-free medium (Invitrogen) containing 1% penicillin/streptomycin (Invitrogen), 1 nM cholera toxin (Sigma-Aldrich), and 1 part of KGF media (MEM Eagle [Spinner modification, Sigma-Aldrich], 5 μg/ml insulin [Sigma-Aldrich], 10 μg/ml transferrin [Sigma-Aldrich], 1.4 μg/ml phosphoetanolamine [Sigma-Aldrich], 10 mM ethanolamine [Sigma-Aldrich], and 0.36 μg/ml hydrocortisone [Calbiochem]). 1% L-glutamine (Invitrogen), 1% penicillin/streptomycin (Invitrogen), 8% chelated FCS (Bio-Rad), 6.6 μg/ml CaCl_2_ (Merck), and 1:1,000 epidermal growth factor (EGF). For coating, a solution containing human placenta collagen (1:40 in PBS) was used.

### Procedures in mice

#### Transcardiac perfusion

Surgical scissors were used to make incisions in the diaphragm and chest to completely expose the heart. A small incision was made on the right atrium, and a perfusion needle was inserted in the left ventricle; thereafter, PBS perfusion was started at a constant speed of ∼1 ml/10 s by manual push of the syringe. A total volume of 30 ml PBS was used for the perfusion of each mouse.

#### Collection of blood

Blood was isolated from mice by cardiac puncture. After a 20-min incubation at RT, blood samples were centrifuged at 20.800 rcf for 15 min. Serum was separated from blood samples and thereafter stored at −80°C.

#### Protein extraction from murine skin and LNs

For most experiments, mice were euthanized, and the ears were shaved to remove hair. Harvested mouse ears were split along the cartilage and cut into small pieces with surgical scissors within 2-ml round bottom Eppendorf tubes. LNs were harvested and placed directly into tubes containing 300 μl tissue extraction buffer (150 mM sodium chloride [NaCl], 50 mM Tris-hydrochloride [Tris-HCl], 1× PIC [Roche]), and one metal bead (Qiagen, 5 mm). The tissues were mechanically disrupted for five times 1 min at 30 Hz on a tissue lyser (Qiagen), centrifuged at 16.900 rcf for 15 min at 4°C, and identical volumes of the supernatant were collected for further analysis. In some cases, specifically for plasminogen expression analysis by ELISA and western blot, as well as in plasmin activity assay, a transcardiac perfusion was performed immediately after euthanasia prior to tissue collection.

#### Elution assay from murine skin explants and LNs

Mice were euthanized, and ears were shaved to remove hair. Ears were harvested and split along the cartilage before placing them with the dorsal side facing down into a 24-well plate containing 300 μl PBS. In the case of subsequent transwell chemotaxis assays, ear skin was placed into a 12-well plate containing 0.9 ml of AIM-V medium and incubated at 4°C on a shaker. After 16–24 h, the supernatant was collected for further analysis.

#### CHS response

CHS-induced inflammation was elicited in two steps as previously described (Arasa et al., 2021b; Vigl et al., 2011). First, mice were anesthetized by inhalation of 2.5% isoflurane and sensitized with 2% oxazolone (Sigma-Aldrich) in acetone:olive oil (4:1) on the shaved abdominal skin (50 μl) and footpads (5 μl). Five to six days later, mice were anesthetized by inhalation of 2.5% isoflurane and challenged with 1% oxazolone in acetone:olive oil (4:1), 10 μl on each side of the ear and/or 10 μl on the footpad of the hind legs. Inflamed tissues or tissue-dLNs were analyzed 24 h later or used for further experiments.

#### TPA

Acute skin inflammation was induced by application of 2 μg of TPA (Sigma-Aldrich) dissolved in 10 μl acetone on the ventral and 10 μl on the dorsal side of the shaved murine ear skin. Inflamed tissues or tissue dLNs were analyzed 24 h later or used for further experiments.

#### *In vivo* treatment of mice with inhibitors of plasmin activation or activity

To inhibit plasmin activation and plasmin activity, mice were treated with a combination of 6 mg/kg mU1 uPA-neutralizing antibody (Lund et al., 2008) and 4 mg/kg plasmin inhibitor C3 (Swedberg et al., 2019). Both compounds were co-administered in 50 μl PBS by intraperitoneal (i.p.) injections at 0 and 12 h of the experiment. CTR groups received PBS only. Mice were euthanized after 24 h.

### Adoptive transfer of DCs

For competitive adoptive transfer studies, LPS-matured WT and CCR7^−/−^ bone marrow–derived DCs were generated as described above and labelled with CSFE (final concentration 5 μM; Invitrogen) or DeepRed dye (final concentration 1 μM; Thermo Fischer Scientific), respectively. Labelling was interchanged in between experiments. Cells were mixed in a 1:1 ratio, and 0.75–1.5 × 10^6^ cells were injected s.c. in 5 μl PBS into the steady-state or CHS-inflamed footpads of WT mice. Footpad-draining popliteal LNs were harvested 18–24 h later, weighed, and passed through a 40-μm cell strainer to generate single-cell suspension. Single-cell suspensions were transferred into 15-ml Falcon tubes, and the volume of the single-cell suspensions was measured. After counting the cellularity (LUNA-FLTM Automated Fluorescence Cell Counter, Logos Biosystems), samples were centrifuged and distributed to 96-well U-bottom plates and incubated with anti-CD16/32 Fc-receptor blocking antibody (clone 93; BioLegend). Staining was performed with the following antibodies: anti-CD11c PE/Cy7 (clone N418), anti-I-A/I-E BV421 (clone M5/114.15.2), anti-CD45 APC/Cy7 (clone 30-F11), and zombie Aqua (all BioLegend). Samples were acquired on a Cytoflex S apparatus (Beckman Coulter) using CytExpert software and analyzed with FlowJo software 10.4.0 (TreeStar).

### FITC painting

Mice were anesthetized by inhalation of 2.5% isoflurane and 20 μl of FITC (Sigma-Aldrich), dissolved at 5 mg/ml in acetone, and dibutyl phthalate (1:1; Sigma-Aldrich) was applied to each side of the ears. 18 h later, the ear-draining auricular LNs were harvested, passed through a 40-μm cell strainer, and the cells were counted. In experiments where further LN digestion was required, auricular LNs were enzymatically digested using DNAseI (100 µg/ml, Sigma-Aldrich), Collagenase P (200 µg/ml, Sigma-Aldrich), Dispase II (800 µg/ml, Sigma-Aldrich) in 50 ml RPMI (Gibco) supplemented with 1% FBS at 37°C for 60–75 min, as previously described (Sokol et al., 2018). Every 10 min, 1 ml of the supernatant was removed and added to cold “quenching buffer” consisting of 2 mM EDTA in RPMI (Gibco) supplemented with 1% FBS. The removed supernatant was replaced with the same volume of fresh enzyme mix. The process was repeated until no tissue fragments remained. LN cell suspensions were stained with PE/Cy7-labelled Armenian hamster anti-mouse CD11c, BV421-labelled rat anti-mouse MHC-II, and APC-labelled rat anti-mouse CD45 (all from BioLegend). Samples were acquired on a Cytoflex S, and the percentage and number of FITC^+^ and FITC^−^ migratory cells were analyzed in FlowJo software 10.4.0 (TreeStar).

### Flow cytometric analysis of uPA, uPAR, and plasminogen levels on LECs

Steady-state or CHS-inflamed ears of WT mice were split along the cartilage and cut into small pieces. The tissue pieces were digested in 4 mg/ml collagenase IV (Thermo Fisher Scientific) in PBS at 37°C for 45 min, passed through a 40-µm cell strainer, and washed with FACS buffer (PBS containing 1% FBS). Single-cell suspensions were stained on ice in FACS buffer according to the following four-step protocol (for 20 min each step, all antibodies from BioLegend unless indicated otherwise): (1) anti-mouse CD16/32 (Fc Block) (clone 93); (2) goat anti-mouse uPAR (polyclonal, AF534; R&D Systems), rat anti-mouse uPA (Clone 901420; R&D Systems), and goat anti-mouse kringle-5 plasminogen (polyclonal, AF742; R&D Systems); (3) donkey anti-rabbit/goat/rat Alexa 488/594/647 (all Invitrogen). (4) Rat anti-mouse CD31 BV421 (clone MEC 13.3; BD Pharmingen), rat anti-mouse CD45 APC/Cy7 (clone 30-F11; BioLegend), Syrian hamster anti-mouse Podoplanin PE/Cy7 (clone 8.1.1; BioLegend), and Zombie Aqua. Samples were acquired on a Cytoflex S apparatus (Beckman Coulter) using CytExpert software and analyzed with FlowJo software 10.4.0 (TreeStar).

For the analysis of LN LECs or immortalized LECs, cells were grown to confluency, washed thoroughly with PBS, lifted from cell culture plates with Accutase (Gibco), and resuspended in FACS buffer. The same, above-mentioned, staining protocol was followed but omitting step (IV) of the protocol.

#### Whole-mount immunofluorescence staining

Mice were sacrificed; the ears were shaved and depilated, harvested, and split into the ventral and dorsal sheets along the cartilage. Next, residual cartilage was removed, and ear halves were placed dermal side down into 24-well TPP plates.

### Intracellular CCL21 whole-mount staining

Dorsal ear sheets were fixed at RT for 2 h in 4% PFA/PBS and washed twice for 30 min with 0.3% Triton X-100/PBS, followed by a 2-h incubation with “immuno-mix” (0.3% Triton X-100/PBS supplemented with 5% donkey serum [Sigma-Aldrich] and 5% BSA [Sigma-Aldrich]). The following primary antibodies were diluted in immuno-mix and added overnight at 4°C: rabbit-anti-mouse LYVE-1 (polyclonal, AngioBio), rat-anti-mouse CD31 (clone MEC 13.3; BD Pharmingen), and goat-anti-mouse CCL21 (polyclonal, R&D Systems). After washing four times with 0.3% Triton X-100/PBS for a total of 2 h, ears were incubated with donkey anti-rabbit IgG-BV421 (BioLegend), donkey anti-rat IgG-AF594, and donkey anti-goat IgG-AF647 (both from Invitrogen) at 4°C for 4 h. Samples were washed again for 2 h with 0.3% Triton X-100/PBS before mounting in Mowiol (Mowiol 4-88; Calbiochem).

### Extracellular CCL21 whole-mount staining

Fresh dorsal ear sheets were incubated for 2 h in 1 ml 0.1% BSA in PBS before adding the following primary antibodies in 0.5 ml 0.1% BSA in PBS overnight at 4°C: rabbit-anti-mouse LYVE-1 (polyclonal, AngioBio), rat-anti-mouse CD31 (clone MEC 13.3; BD Pharmingen), and goat-anti-mouse CCL21 (polyclonal, R&D Systems). After washing four times with 1 ml PBS for a total of 2 h, ears were incubated with the following secondary antibodies in 0.5 ml 0.1% BSA in PBS at 4°C for 4 h: donkey anti-rabbit IgG-BV421 (BioLegend), donkey anti-rat IgG-AF594, and donkey anti-goat IgG-AF647 (both Invitrogen). Samples were washed again four times for a total of 2 h with 1 ml PBS before mounting in Mowiol (Mowiol 4-88; Calbiochem).

### Imaging of whole-mount preparations

Imaging of whole-mount preparations was performed on a Zeiss LSM 880 inverted confocal microscope, equipped with the following objectives: 10× 0.3 NA, enhanced contrast (EC) Plan-Neofluar and 20× 0.8 NA Plan-Apochromat, or Zeiss LSM 780 upright confocal microscope, equipped with the following objectives: 10× 0.3 NA EC Plan-Neofluar Ph1 M27 and 20× 0.8 NA Plan-Apochromat M27 (all Carl Zeiss Microscopy). Images were acquired using ZEN 2012 software, and staining intensities were quantified with FIJI (ImageJ, 1.52p, [Schindelin et al., 2012]) in a blinded manner.

### CCL21 gradient analysis

Intensities of interstitial CCL21 signals were quantified on maximum intensity projections ranging between 20 and 40 µm z-stacks with a z-step size of 1 µm. Distance gradients were computed using custom-written scripts in MATLAB, similar to as described previously (Holtkamp et al., 2021). In brief, first, capillaries of lymphatics were manually outlined by an experimenter blinded to the experimental condition. The results were converted into binary masks from which a Euclidean distance transform was computed to result in a distance gradient map, containing the shortest Euclidean distance of each non-masked pixel to the closest LV in pixel units, respectively. Using the pixel resolution of the acquired microscopy images, distance values were transformed into metric space in µm. The Euclidean distance transform masks were first rounded to integers in µm and then applied to the microscope images. Next, fluorescence intensities of pixels with the same integer distance were averaged to result in one distance gradient vector per image, containing the average fluorescence intensity per Euclidean distance from lymphatics in µm, and finally averaged across biological replicates per condition. Five images were taken from different locations within one mouse ear and averaged as one biological replicate. Samples were normalized to the CCL21 intensity at the WT vessel (0 µm) (or the uPA^mut^ vessel (0 µm) in the case of the heparitinase treatment performed—see below. Isotype stainings were taken as negative CTRs.

### Heparitinase treatment

UPA^mut^ mice were sacrificed; the ears were shaved and depilated, harvested, and split into the ventral and dorsal sheets along the cartilage, and cartilage debris was removed. Next, ear halves were placed dermal side down into 24-well TPP plates containing 50 U/ml of heparitinase II (Sigma-Aldrich) in 1 ml RPMI (Gibco) and incubated for 2 h at 37°C. Subsequent blocking and staining were performed as described above.

### Analysis of CD11c^+^CD45^+^ DCs in whole mounts

Dorsal ear sheets were fixed at RT for 2 h in 4% PFA/PBS and washed twice for 30 min with PBS. The following directly coupled antibodies were diluted in PBS and added for 1 h: Armenian hamster anti-mouse CD11c PE (clone N418; BioLegend), rat anti-mouse LYVE-1 AF488 (clone ALY7; eBioscience), rat anti-mouse CD45 AF700 (clone 30-F11; BioLegend), and rat anti-mouse CD31 BV421 (clone MEC13.3; BD Pharmingen). Samples were washed again twice for 30 min with PBS before mounting with Mowiol (Mowiol 4-88; Calbiochem). Imaging of whole-mount preparations was performed on a Leica TCS SP8 inverted confocal microscope, equipped with the following objectives: 10× 0.3 NA PH1 HC PL FLUOTAR, 20× 0.7 NA PH2 HC PLAN APO, 20× 0.7 NA Oil/W/Glyc HC PLAN APO, 25× 0.95 NA L Water HCX IRAPO, 40× 1.1 NA Water HC PL IRAPO CORR, and 63× 1.4 NA Oil CS2 HC PL APO (all Leica Microsystems). 4–7 images were analyzed per ear. Images were acquired blindly using Leica LASX SP8 software. Localization and quantification of CD11c^+^CD45^+^ cells were performed with the Imaris software (Bit Plane) in a blinded manner.

### Immunofluorescence-based quantification of FITC^+^ migratory DCs in LNs

Auricular LNs from adult WT and uPA^mut^ mice were harvested following FITC painting and embedded in OCT compound (Sakura Finetek). Freshly cut 10-µm cryosections were fixed for 10 min in 4% PFA, washed with TBS-T (0.3% Tween-20), and blocked for 1 h at RT in 3% BSA/0.1% Triton X-100 in PBS. Sections were incubated overnight at 4°C with rabbit anti-Lyve-1 (polyclonal, AngioBio) and Armenian hamster anti-CD11c (clone N418; Thermo Fisher Scientific) diluted in blocking buffer. The next day, slides were washed three times with PBS, blocked with 5% goat serum/0.1% Triton X-100 in PBS for 30 min at RT, and then incubated with secondary antibodies (goat anti-rabbit Alexa Fluor 594; goat anti-Armenian hamster Alexa Fluor 647; Invitrogen) and DAPI nuclear stain in 2% goat serum/0.1% Triton X-100 in PBS for 1–3 h at RT in a dark humidified chamber. Finally, slides were washed three times with PBS, mounted with Mowiol 4-88 (Sigma-Aldrich), and imaged on a Vectra Polaris slide scanner (Akoya Biosciences) using a 20× objective and a Hamamatsu C11440-50 Series camera. Multispectral images were acquired using PhenoImager HT software and unmixed and exported using INFORM software (v3.2.2, Akoya Biosciences). For each mouse LN, 3–6 representative images were acquired from different tissue depths, yielding a total of 61 images from 13 mice. Spectrally unmixed images were then analyzed using QuPath software (v0.5.1).

The cell segmentation procedure was adapted from a previously described protocol (Franken et al., 2024). Regions of interest were defined for each image, and tissue compartments were classified in QuPath (v0.5.1) using the built-in trainable pixel classifier to distinguish SCS, LN parenchyma, and background, with Lyve-1 staining used to differentiate SCS from parenchyma. Cell segmentation was performed using “StarDist” (Schmidt et al., 2018), a deep learning–based nucleus detection algorithm, applied to the DAPI channel. Segmented cells were subsequently assigned to their corresponding tissue compartments using the trained object classifier. Single-cell data (cell counts, densities, and spatial coordinates, as well as per-cell marker intensities) were exported from QuPath as .csv files. Data were processed in RStudio (v2024.12.1). Using the flowCore package (Bioconductor) the .csv files were imported, converted into flow cytometry standard (.fcs) format. The obtained FCS files were imported into FlowJo (v5, BD Biosciences) for gating and visualization. The gating strategy included: (1) all cells (based on DAPI positivity and cell area), (2) CD11c^+^ cells (CD11c–Alexa Fluor 647 with DAPI), and (3) FITC^+^ cells (FITC–Alexa Fluor 488 with DAPI). Gates were defined using cells from marker-negative areas to establish fluorescence thresholds (Fig. S5 J). Statistical analyses were performed as described below.

### Extracellular CCL21 staining in LN sections

Inguinal LNs of adult (6–10 wk) WT and uPA^mut^ mice were embedded in OCT. Unfixed, freshly cut 10-μm sections were blocked with 5% donkey serum (D9663; Sigma-Aldrich) in PBS and stained immediately with goat anti-mouse CCL21 (AF457; R&D Systems, 13 μg/ml), rabbit anti-mouse LYVE-1 (polyclonal, AngioBio), and rat anti-mouse B220/CD45R (BD Pharmingen) in blocking solution for 1 h at RT, as previously described (Ulvmar et al., 2014). After a 10 min PBS wash, sections were incubated with secondary antibodies donkey anti-goat IgG-AF549, donkey anti-rabbit IgG-AF647, donkey anti-rat IgG-AF488, and Hoechst (all from Invitrogen) in blocking solution for 1 h at RT. After further PBS washing, sections were mounted with Mowiol (Mowiol 4-88; Calbiochem) and imaged on a slide scanner Akoya Vectra Polaris (Akoya Biosciences) with a 20× magnification and a Hamamatsu C11440-50 Series camera.

Multispectral images were acquired using PhenoImager HT software and analyzed using INFORM 2.6.0 (Akoya Biosciences), a machine learning–based trainable tissue segmentation tool. All stained slides were incorporated into the same INFORM project. Here, three different tissue categories were defined, based on the LYVE-1 and B220 staining, and trained on: “SCS” (i.e., tissue space between the LN border the LYVE-1^+^ SCS floor), “B cell follicle” (defined as B220^+^ area), and “T cell area” defined as nodal tissue not belonging to the SCS or B cell follicle. The CCL21 signal intensity data was combined with the tissue segmentation to allow downstream normalization of signal intensity to the area of each tissue category, i.e., SCS and T cell area. Exported data were analyzed using RStudio (Version 2023, Posit).

### Western blot analysis of murine CCL21 cleavage

Western blot analysis of *in vivo–* and *in vitro*–derived protein samples was performed on Vertical Electrophoresis system (Bio-Rad, GenScript) with Stain-Free Precast Gels with a polyacrylamide gradient of 4–20% (BioRad, GenScript). In brief, samples were denatured by addition of 4× reducing Laemmli Sample Buffer (Bio-Rad) to a final concentration of 1×, prior to heating at 95°C for 5 min. Samples and protein ladder (Precision Plus Protein Dual Color, Bio-Rad) were loaded on gels, and electrophoresis was conducted for up to 2:45 h at 60 V. Protein transfer to a nitrocellulose membrane (0.2 μm, Bio-Rad) was performed as described by the manufacturers’ guidelines for 20–40 min at 19 V on a Bio-Rad Trans-Blot SD Semi-Dry Transfer Cell.

Membranes were incubated in 5% milk powder in PBS to block unsaturated protein-binding sites. The following primary antibodies were added in 5% milk powder in PBS: rabbit anti-mouse CCL21 (polyclonal, PeproTech), rabbit anti-human CCL21 (polyclonal, PeproTech), or goat anti-mouse kringle 5 plasminogen (AF742-SP; R&D Systems, polyclonal). Membranes were washed four times for 3 min each with 0.1% Tween20/PBS before adding secondary antibodies in 5% milk powder in PBS: donkey anti-rabbit IgG-HRP (Invitrogen) or rabbit anti-goat IgG-HRP (Invitrogen). Membranes were washed once again four times for 3 min each and afterward incubated for 5 min with Clarity Western ECL Substrate (BioRad) before imaging with a ChemiDoc MP Imaging System (BioRad). Western blot band intensities were quantified with the Image Lab software (V 6.0.1, Bio-Rad).

### *In vitro* CCL21 cleavage by recombinant plasmin

Murine recombinant CCL21 (#250-13; PeproTech) and recombinant murine plasmin (Molecular Innovations) at molar ratios of CCL21 to plasmin of 1:0.02–1:0.16 (starting with 100 nM CCL21 and 2 nM plasmin) were incubated in PBS or serum-free ProCHO medium (Lonza) for 4 h at 37°C. Reactions were stopped by addition of 4× reducing Laemmli Buffer (Bio-Rad). Samples were heated to 95°C for 2 min, followed by western blot analysis. Alternatively, a fixed molar ratio of CCL21:plasmin = 100 nM:8 nM was incubated for 0, 5, 10, 15, 30, 60, 120, 180, and 240 min at 37°C prior to addition of 4× reducing Laemmli Buffer, followed by heating to 95°C for 2 min and subsequent western blot analysis. In some cases, reactions were stopped by addition of the plasmin inhibitor “C3” at the indicated concentrations. The same experimental setup, with identical ratios and starting concentrations, was applied for recombinant human CCL21 (#300-35A; PeproTech) and recombinant human plasmin (Molecular Innovations).

### Western blot of recombinant human CCL21 and CCL21-ΔC

For cloning of human CCL21-ΔC, corresponding to the amino acids 1–79 of mature CCL21, the previously published His6-SMT3-CCL21trunc (amino acids 1–74; [Hauser et al., 2016]) was amplified using the following primers: forward 5′-GGG AAT TGT GAG CGG ATA ACA ATT CCC CTC TAG AAA TAA TTT TG-3′ and reverse 5′-GGT GCT CGA GTC AGC CCT GGG CTG GTT TCT GTG GGG ATG GTG TCT TG-3′ inserting the missing five amino acids at the C terminus. Furthermore, XhoI and XbaI restriction enzymes were used to ligate His6-SMT3-CCL21-ΔC into the corresponding cutting sites of pSUMO. Subsequently, CCL21-ΔC was expressed and purified as previously described (Artinger et al., 2021). In brief, pSUMO His6-SUMO-hCCL21-ΔC was transformed and expressed in *Escherichia coli* BL21(DE3) RIPL at 37°C for 5 h after induction with 1 mM IPTG. Subsequently, bacterial cell pellets were dissolved and lysed in AFC lysis buffer (50 mM sodium phosphate, 300 mM NaCl, 10 mM imidazole, 2 mM tris(2-carboxyethyl)phosphine, and 1 mM PMSF, pH 8.0) before dissolving inclusion bodies in lysis buffer containing six guanidine HCl. Human chemokines were then separated from bacterial protein contaminants via immobilized metal ion chromatography and eluted by pH reduction before infinite dilution in refolding buffer (50 mM Tris, 150 mM NaCl, 10 mM L-cysteine, 0.5 mM L-cystine, 1 mM EDTA, 1 mM PMSF, and 10% glycerol, pH 8.5). Furthermore, native chemokine N termini were retrieved by Ulp1 protease cleavage of the His6-Sumo purification tag and additional cation ion exchange chromatography (CIEX) in CIEX buffer (100 mM Tris, pH 8.0). Ultimately, correctly folded and biologically active chemokines were polished via C18 reverse phase HPLC, lyophilized, and stored at −20°C.

### Western blot of human skin

The use of human samples for research purposes was approved by the local ethics commission Maasstad Hospital, Rotterdam, The Netherlands (study protocol number 55149.101.15.). Informed consent was obtained from all subjects involved in the study. Fat was removed from skin biopsies. Biopsies were cut into small fragments using surgical scissors and placed in tubes containing 300 μl tissue extraction buffer (150 mM NaCl, 50 mM Tris-HCl, 1× PIC [Roche]), and one metal bead (Qiagen, 5 mm). The tissues were mechanically digested for five times 1 min at 30 Hz on a tissue lyser (Qiagen), centrifuged at 16.900 rcf for 15 min at 4°C, and identical volumes of the supernatant were collected for further analysis. Gel electrophoresis and western blot were performed as described previously. For the detection of human CCL21, rabbit anti-human CCL21 (polyclonal, PeproTech) primary antibody and donkey anti-rabbit IgG-HRP (Invitrogen) secondary antibody were used.

### ELISA-based plasminogen quantification

The Mouse Plasminogen Total ELISA Kit (ab198511; Abcam) was used according to the manufacturer’s instructions. Briefly, protein extracts from ears and dLNs of CHS-inflamed and non-inflamed mice were diluted 1:50 in blocking buffer and added in 96-well flat-bottom wells. The plate was shaken at 300 rpm for 30 min and then washed three times with 1× wash buffer. Next, the plasminogen primary antibody was added to the wells, and the plate was shaken at 300 rpm for 30 min. The wells were washed three times with 1× wash buffer, and HRP secondary antibody was added, and the plate was shaken at 300 rpm for 30 min. The wells were washed three times with 1× wash buffer, tetramethylbenzidine (TMB) substrate was added to the wells for 1–5 min, and the reaction was quenched by the addition of 1 N sulfuric acid (H_2_SO_4_). The intensity of the signal at 450 nm was determined with an Infinite M200 PRO plate reader (Tecan). Plasminogen concentration in each sample was determined from the standard curve using GraphPad Prism software.

### *In vitro* plasmin activity assay

For the quantification of plasmin activity in CTR or CHS-inflamed tissue protein extracts, the plasmin activity kit (ab273301; Abcam) was used according to the manufacturer’s instructions. In brief, protein extracts from ears and dLNs of CHS-inflamed and non-inflamed mice were mixed with the assay buffer in 96-well flat-bottom wells, with a see-through bottom, and the substrate was added just prior to starting the assay. The absorbance was recorded at 405 nm in a kinetic mode at 37°C every 30 s for a total of 30 min using an Infinite M200 PRO plate reader (Tecan). Unit definition: One unit of plasmin activity is the amount of enzyme that releases 1.0 μmol of para-nitroaniline per min at pH 8.4 at 37°C. Data are shown as absolute plasmin activity per analyte.

For the evaluation of the inhibitory effect of C3 on plasmin activity, the plasmin activity kit (MAK 244-1KT; Sigma-Aldrich) was used according to the manufacturer’s instructions. In brief, recombinant plasmin (12 μM, Molecular Innovations) with or without the addition of inhibitors PIC (1×, Roche) or C3 (200 or 500 nM) were mixed with the assay buffer in 96-well flat-bottom wells with a see-through bottom, and the substrate was added just prior to starting the assay. The increase in fluorescence was recorded in a kinetic mode at 37°C every 30 s for a total of 30 min using an Infinite M200 PRO plate reader, and the signal intensity was normalized to time point zero (t0).

### Cell-based cleavage of CCL21 *in vitro*

For LEC-based cleavage assays, LN LECs or immortalized LECs were seeded at 25,000 cells/well and keratinocytes at 50,000 cells/well into pre-coated 48-well TPP cell culture plates and grown to confluency in each cell type´s optimal growth medium overnight. Cells were washed three times with pre-warmed PBS and incubated for 2 h in Pro-Cho serum free medium (for analysis of LEC-induced cleavage) or AIM-V medium (if supernatant was used for 3D collagen migration assays). Importantly, serum-free medium was used since serum contains endogenous plasmin inhibitors. Cells were washed again three times with pre-warmed PBS, and treatment conditions were added in 150 μl Pro-Cho or AIM-V medium. For assays involving mU1-Urokinase neutralizing antibody, the cells were pre-blocked in 100 μl Pro-Cho medium/AIM-V medium containing the indicated concentrations of mU1, and further treatments were added in 50 μl medium directly to the pre-blocked wells (total volume of 150 μl). Cells were further treated with 100 nM recombinant murine CCL21 (PeproTech) and 20 nM recombinant murine plasminogen (Molecular Innovations). Keratinocytes were treated with 10 nM or 20 nM recombinant murine plasminogen. Inhibition of CCL21 cleavage was induced by addition of mU1 uPA-neutralizing antibody (20 µg/ml) or the plasmin inhibitor C3 (500 nM) or a 1× solution of the PIC (Roche). For investigation of CCL21 cleavage, cells were incubated for 4 h at 37°C. For investigation of plasminogen to plasmin conversion, cells were incubated for a total of 72 h, and samples were taken at 4, 24, 48, and 72 h. At the indicated time points, the supernatant was collected, and 4× reducing Laemmli Buffer (Bio-Rad) was added, followed by heating to 95°C for 2 min and subsequent western blot analysis.

For DC-based cleavage assays, bone marrow–derived DCs were seeded at 60,000 cells/well in a 48-well plate with serum-free DC medium containing 100 nM recombinant murine CCL21 (PeproTech) and 20 nM recombinant murine plasminogen (Molecular Innovations) and incubated for a total of 10 h at 37°C. Samples were taken at 24 h, and 4× reducing Laemmli Buffer (Bio-Rad) was added, followed by heating to 95°C for 2 min and subsequent western blot analysis.

For keratinocyte-based cleavage assays, keratinocytes were seeded at 40,000–75,000 cells/well in a 48-well plate with serum-free keratinocyte medium containing 100 nM recombinant murine CCL21 (PeproTech) and 20 nM recombinant murine plasminogen (Molecular Innovations) and incubated for a total of 4 h at 37°C. Samples were taken at 24 h and 4× reducing Laemmli Buffer (BioRad) was added, followed by heating to 95°C for 2 min and subsequent western blot analysis.

### 3D collagen DC migration assay

A scaled-up version of the LEC-based CCL21 cleavage assays was performed in 12-well TPP plates, seeding an initial number of 100,000 immortalized LECs and scaling up all reagents according to the increased cell culture volume of 450 μl. After 4 h, the supernatants were supplemented with 10% FBS and 500 nM C3 to inhibit further CCL21 cleavage, snap frozen, and stored at −20°C until further use. 3D collagen chemotactic cell migration assays were performed in Ibidi µ-slide chambers (Cat. No: 80326), in accordance with the manufacturer’s instructions (Ibidi) and as previously described (Purvanov et al., 2018). In brief, 20 μl 10× DMEM, 10 μl 7.5% NaHCO_3_, 2 μl 10 N NaOH, and 150 μl of a 3% PureCol collagen I solution (Advanced BioMatrix) were premixed, then 90 μl cell suspension (1 × 10^7^ LPS-matured BMDCs/ml stained with cell tracker Green CMFDA, Invitrogen) were added, mixed carefully, and placed into the middle channel of the chemotaxis slide. After polymerization for 30–45 min in a humidified 37°C, 5% CO_2_ incubator, the source and the sink tanks of the chamber were filled according to the manufacturer’s instructions. Chemotactic stimuli comprised recombinant human proteins or supernatant of *in vitro* microenvironment assays, which had been supplemented with 10% FCS and 500 nM C3 to inhibit additional CCL21 cleavage. Migration of the cells was imaged live using the time-lapse microscope Zeiss Axiovert 200M equipped with an AxioCam MRm camera and an automated stage and a temperature- and humidity-controlled stage-top Tokai Hit INU incubator (Shizuoka Japan) for 4 h with intervals of 5 min. Motile cells at the center of the migration chamber that displaced an accumulated distance of >50 μm during the recording (∼20–40 cells per field of view) were manually tracked using ImageJ (Fiji). Directionality (total track length over track displacement length), yFMI (directionality along the y axis = direct axis between the two stimuli), and Euclidian distance (distance from origin to endpoint) were calculated for each cell using the “Chemotaxis and Migration Tool” plugin (Ibidi).

### ELISA for CCL21 quantification

Clear flat-bottom Immuno non-sterile 96-well plates (Thermo Fisher Scientific) were coated with capture antibody (polyclonal goat anti-mouse CCL21 AF457; R&D Systems), diluted at 2 μg/ml in PBS, and incubated at 4°C overnight. Plates were washed three times with PBS/Tween (0.1%), blocked with 2% Milk/PBS for 2 h at RT, and washed three times with PBS/Tween (0.1%) before chemokine standards (100–0.0975 ng/ml), and samples diluted 1:2 (serum, LN and ear eluates and extracts) were added and incubated for 1 h at RT. The detection antibody (for full-length and cleaved CCL21: biotinylated goat anti-mouse CCL21 BAF457 with a detection limit of 122 pg/ml; R&D Systems, for full-length CCL21 only: rat anti-mouse CCL21 MAB457 with a detection limit of 900 pg/ml; R&D Systems) was diluted at 1 µg/ml in 2% milk/PBS, incubated for 1 h at RT, and washed five times with PBS/Tween (0.1%). Streptavidin-HRP (BioLegend) or donkey anti-rat IgG-HRP (Thermo Fisher Scientific) was diluted to 1 µg/ml in 2% Milk/PBS and added to each well. The plates were incubated for 1 h at RT and washed five times with PBS/Tween (0.1%), followed by five times with PBS. The reaction was developed by the addition of TMB substrate (BioLegend), prepared as per manufacturer’s instructions. It was developed for up to 10 min in the dark until stopped by the addition of 1 M H_2_SO_4_. The intensity of the signal at 450 nm was determined with an Infinite M200 PRO plate reader (Tecan). CCL21 concentration in each sample was determined from the standard curve using GraphPad Prism software.

### Quantitative PCR analysis

Total RNA was isolated using 1 ml TRIzol (Ambion) per 10-cm cell culture plate of 80% confluent LN LECs, according to manufacturer’s protocol. cDNA was synthesized from isolated RNA using the RQ1 RNase-Free DNase (Promega) and the High-Capacity cDNA Reverse Transcription Kit (Thermo Fisher Scientific) according to manufacturer’s instructions. Quantitative PCR (qPCR) was performed with PowerUp SYBR Green Master Mix (Thermo Fisher Scientific). Relative expression was calculated with the 1/2^ΔCT^ method, where delta cycle threshold (ΔCT) = Ct_Target_−Ct_18s_, and with the 2^−ΔΔCT^ method, where delta–delta cycle threshold (ΔΔCT) = ΔCt_uPAmut/KO_−ΔCt_WT_. The following primers were used (all from Microsynth): *18 s* forward: 5′-AGG​AAT​TCC​CAG​TAA​GTG​CG-3′ and reverse: 5′-GCC​TCA​CTA​AAC​CAT​CCA​A-3′; *PLGRKT* forward: 5′-CGG​AAC​TCT​CCT​ACA​GAG​AAT​GA-3′ and reverse: 5′-TTG​GCA​GCT​CCA​GCT​TCG​TC-3′; *PLAU* forward: 5′-GCG​CCT​TGG​TGG​TGA​AAA​AC-3′ and reverse: 5′-TTG​TAG​GAC​ACG​CAT​ACA​CCT-3′; *PLAUR* forward: 5′-GGC​TTA​GAT​GTG​CTG​GGA​AA-3′ and reverse: 5′-CAA​TGA​GGC​TGA​GTT​GAG​CA-3′; *CCL19* forward: 5′-TCG​TGA​AAG​CCT​TCC​GCT​ACC​T-3′ and reverse: 5′-CAG​TCT​TCG​GAT​GAT​GCG​ATC​C-3′; *CCL21* forward: 5′-GGG​TCA​GGA​CTG​CTG​CCT​TAA​G-3′ and reverse: 5′-AGC​TCA​GGC​TTA​GAG​TGC​TTC​C-3′.

### Chemotaxis assays

LPS-matured bone marrow–derived DCs were prepared as described above. For competitive Transwell studies, WT and CCR7^−/−^ DCs were harvested and labelled for 10 min in PBS with CSFE (final concentration 5 µM) (Invitrogen) or DeepRed dye (final concentration 1 µM) (Thermo Fischer Scientific), respectively. Labelling colors were interchanged in between experiments. Cells were left to rest in a humidified incubator at 37°C and 5% CO_2_ for 30 min in medium after labelling. Subsequently, dead cells were removed by centrifugation over a FBS gradient and mixed in a 1:1 ratio in AIM-V medium supplemented with 2% FCS, and 100,000 cells were added in the top well of a 5-µm pore-size transwell insert (Jet Biofil) and left to migrate toward the bottom well at 37°C for 2 h. Chemotactic stimuli in the lower well consisted of 100 ng/ml murine CCL21 (PeproTech) or medium from *ex vivo* elution assays of mouse ear skin, which had been supplemented with 2% FCS. In some experiments, DCs were added in the top well together with 20 nM murine plasminogen (Molecular Innovations) or with 8 nM murine plasmin (Molecular Innovations) chemotactic stimuli consisting of 100 ng/ml human CCL21 (PeproTech), 100 ng/ml human CCL21-ΔC (generated as described above), 100 ng/ml murine CXCL12 (PeproTech), 100 ng/ml murine CXCL12 and 20 nM murine plasminogen (Molecular Innovations), 100 ng/ml murine CXCL12, and 8 nM murine plasmin (Molecular Innovations). In some cases, 1 µg/ml of CCL21 blocking antibody (polyclonal, AF457; R&D Systems) was added to the lower wells. After 2 h, transmigrated DCs were stained with Armenian hamster anti-mouse CD11c PE/Cy7 (clone N418) and rat anti-mouse MHC-II BV421 (clone M5/114.15.2; both BioLegend), quantified by flow cytometry on a Cytoflex S apparatus (Beckman Coulter) using CytExpert software, and analyzed with FlowJo software 10.4.0 (Treestar).

### Transmigration assay

Transwells (5 µm pore size, Jet Biofil) were coated with collagen (10 μg/ml; Advanced BioMatrix) and fibronectin (10 μg/ml; Millipore), and 50,000 purified LN LECs were added into the top well of each insert in 100 μl of LN LEC medium. The lower well of each insert was filled with 700 μl LN LEC medium, and cells were incubated for 2 days at 37°C and 5% CO_2_. Transmigration assays were performed by adding 10,000 LPS-matured YFP^+^ WT DCs, generated from the bone marrow of CD11c-YFP mice (Lindquist et al., 2004), to the top well in 100 μl AIM-V medium supplemented with 2% FBS in presence or absence of either 20 nM murine plasminogen (Molecular Innovations) or of 8 nM murine plasmin (Molecular Innovations). DCs were left to migrate toward the bottom well at 37°C for 4 h. Chemotactic stimuli in the lower well consisted of 100 ng/ml murine CCL21 (PeproTech), 100 ng/ml human CCL21 (PeproTech), 100 ng/ml human CCL21-ΔC (generated as described above), 100 ng/ml murine CXCL12 (PeproTech), 100 ng/ml murine CXCL12 and 20 nM murine plasminogen (Molecular Innovations), 100 ng/ml murine CXCL12 and 8 nM murine plasmin (Molecular Innovations), or AIM-V medium alone. Please note that concentrations of CCL21 and plasminogen were identical to the ones used in the *in vitro* LEC cleavage assay of CCL21. Each condition was performed in four technical replicates. After 4 h, transmigrated GFP^+^ DCs were quantified by flow cytometry on a Cytoflex S apparatus (Beckman Coulter) using CytExpert software and analyzed with FlowJo software 10.4.0 (Treestar).

### DC crawling assay

Channelled chamber slides (μ-Slide VI0.4, IBIDI) were coated with collagen (10 μg/ml; Advanced BioMatrix) and fibronectin (10 μg/ml; Millipore), and cultured LN LECs (60,000 cells in LN LEC medium) were seeded into the channel and left to adhere and from monolayers for 4 days. On the day of the assay, 30,000 LPS-matured YFP^+^ WT DCs, generated from the bone marrow of CD11c-YFP mice (Lindquist et al., 2004), were added on top of the monolayers in LN LEC media in presence or absence of either 20 nM murine plasminogen (Molecular Innovations) or of 8 nM murine plasmin (Molecular Innovations). As a positive CTR, monolayers were pre-blocked with 5 μg/ml ROCK-inhibitor Y27632 (Nitschke et al., 2012) in 30 μl LN LEC media for 30 min followed by the addition of 30,000 LPS-matured YFP^+^ WT DCs, together with 5 μg/ml ROCK-inhibitor in 30 μl LN LEC starvation media (without FBS). After 20 min, chambers were rinsed twice with starvation media to remove nonadherent DCs. After a further 10 min equilibration at 37°C, time-lapse imaging was performed on a fluorescent microscope (Nikon Eclipse Ti-E) equipped with a Hamamatsu ORCA-Flash4.0 CCD camera (Hamamatsu, Japan) and a 20× objective (NA: 0.75, Nikon). Phase contrast and fluorescence images in the FITC channel were captured every 30 s for 30 min using NIS-Elements software. Movies were analyzed using IMARIS software (Bit Plane), as previously described (Teijeira et al., 2017). For semiautomated cell tracking, tracks were generated by an automatic algorithm and verified manually. Only tracks of cells recorded for >10 min and with a total path length of >60 μm were included in the analysis. Analysis criteria were calculated as follows: speed = total track length/track duration; chemotactic index = displacement/length.

### Statistical analysis

Results are presented as mean ± SEM. Statistical analysis was performed, and graphs were created with Prism 8 software (GraphPad Software Inc.). For all data sets, data distribution was assumed to be normal, but this was not formally tested. The Null-hypothesis was rejected when P < 0.05. Parametric two-tailed unpaired Student’s *t* test was used to compare two groups of samples, and parametric two-tailed paired Student’s *t* test was applied if samples originated from the same animal or experiments from different dates were compared. For multiple comparisons, the data sets were analyzed using non-paired ordinary one-way ANOVA, followed by Tukey correction with single pooled variance. Whole-mount analysis and crawl-in data comparing effects on different days over two treatment groups were analyzed by two-way ANOVA using multiple comparisons, followed by a Tukey correction. Microscopy images were quantified in a blinded manner.

### Online supplemental material

Fig. S1 shows the *in vitro* CCL21 cleavage activity of human and murine plasmin. Fig. S2 shows the flow cytometry–based analysis of uPAR, uPA, and plasminogen expression on leukocytes and stromal cells in murine skin. Fig. S3 provides further information on the expression of uPA/uPAR/plasminogen in primary LN LECs as well as the CCL21 cleavage ability of DCs and keratinocytes. Fig. S4 shows *in vitro* assays performed to assess the CCL21 cleavage-independent effects of plasminogen and plasmin on DC migration. Fig. S5 comprises additional data from FITC painting experiments and shows the impact of CHS on immobilized CCL21 levels in dLNs. Table S1 summarizes the technical specifications of all primary and secondary antibodies used in this study. Videos 1 and 2 shows intravital time-lapse imaging performed in the ear skin of *Prox-1 mOrange2* xCD11cYFP mice to visualize DC presence and movement in the dermis and within lymphatics under CTR (Video) and CHS-inflamed (Video 2) conditions. Video 3 shows fluorescently labelled DCs crawling a 3D collagen migration assay (see Fig. S4, H and I). Video 4 shows CD11cYFP DCs crawling over an LEC monolayer (see Fig. S4, G and H).

## Supplementary Material

Table S1 shows list of antibodies.

SourceData F3 is the source file for Fig. 3.

SourceData F4 is the source file for Fig. 4.

SourceData F8 is the source file for Fig. 8.

SourceData FS1 is the source file for Fig. S1.

SourceData FS3 is the source file for Fig. S3.

## Data Availability

Raw data will be made available on the ETH research collection (public repository) after acceptance of the manuscript. URL: https://doi.org/10.3929/ethz-c-000787623.
