# Supplementary material for: uPA-mediated remodeling of CCL21 gradients regulates lymphatic migration of dendritic cells
Source: J Cell Biol. 2026 Jan 27;225(3):e202412190. doi: 10.1083/jcb.202412190 (PMC12839967; doi:10.1083/jcb.202412190)
Supplement: Table S1 — shows list of antibodies. [file jcb_202412190_tables1.docx]

**Table 1. List of antibodies**

| **Primary antibody: FACS** | **Company and cat. No.** | **Dilution** | **Starting Conc. Mg/ml** | **Final Conc. Ug/ml** |
| --- | --- | --- | --- | --- |
| rat anti-mouse CD31 (MEC 13.3, APC) | BioLegend, 102501 | 1/200 | 0.2 | 1 |
| syrian hamster anti-mouse PDPN (8.1.1, PE/CY7) | BioLegend, 127412 | 1/200 | 0.2 | 1 |
| armenian hamster anti-mouse CD11c (N418, PE/Cy7) | BioLegend, 117317 | 1/200 | 0.2 | 1 |
| rat anti-mouse MHC-II (M5/114.15.2 BV421) | BioLegend, 107632 | 1/500 | 0.2 | 0.4 |
| rat anti-mouse CD16/32 (93) | BioLegend, 101301 | 1/50 | 0.5 | 10 |
| goat anti-mouse uPAR | R&D Systems, AF534 | 1/200 | 0.2 | 1 |
| rat anti-mouse uPA (901420) | R&D Systems, MAB9185 | 1/200 | 0.2 | 1 |
| goat anti-mouse kringle-5 plasminogen | R&D Systems, AF742 | 1/200 | 0.2 | 1 |
| rat anti-mouse CD31 (MEC13.3, BV421) | BD Pharmingen, 562939 | 1/200 | 0.2 | 1 |
| rat anti-mouse CD45 (30-F11, APC/Cy7) | BioLegend, 103115 | 1/200 | 0.2 | 1 |
| rat anti-mouse CD45 (30-F11, APC) | BioLegend, 103111 | 1/200 | 0.2 | 1 |

| **Secondary antibody: FACS** | **Company and Cat. No** | **Dilution** | **Starting Conc. Mg/ml** | **Final Conc. Ug/ml** |
| --- | --- | --- | --- | --- |
| donkey anti-rabbit IgG-AF488 | Invitrogen, A-21206 | 1/300 | 2.0 | 6.6 |
| donkey anti-goat IgG-AF594 | Invitrogen, A-11058 | 1/300 | 2.0 | 6.6 |
| donkey anti-rat IgG-AF647 | Invitrogen, A78947 | 1/300 | 2.0 | 6.6 |

| **Primary antibody: Immunostaining** | **Company and Cat. No** | **Dilution** | **Starting Conc. Mg/ml** | **Final Conc. Ug/ml** |
| --- | --- | --- | --- | --- |
| rabbit anti-mouse LYVE-1 | Angiobio, 11-034 | ears 1/200  LNs 1/100 | 1.0 | 5 / 10 |
| rat anti-mouse CD31 (MEC 13.3) | BD Pharmingen, 550274 | 1/100 | 15.625 | 156.25 |
| goat anti-mouse CCL21 | R&D Systems, AF457 | ears 1/50 LNs 1/15 | 0.2 | 4 / 13.3 |
| armenian hamster anti-mouse CD11c (N418, PE) | BioLegend, 117307 | 1/200 | 0.2 | 1 |
| rat anti-mouse LYVE-1 (ALY7, AF488) | eBioscience, 53-0443-80 | 1/200 | 0.5 | 2.5 |
| rat anti-mouse CD45 (30-F11, AF700) | BioLegend, 103128 | 1/200 | 0.5 | 2.5 |
| rat anti-mouse CD31 (MEC13.3, BV421) | BD Pharmingen, 562939 | 1/200 | 0.2 | 1 |
| rat anti-mouse B220/CD45R | BD Pharmingen, 553084 | 1/100 | 0.5 | 5 |

| **Secondary antibody: Immunostaining** | **Company and Cat.No** | **Dilution** | **Starting Conc. Mg/ml** | **Final Conc. Ug/ml** |
| --- | --- | --- | --- | --- |
| donkey anti-rabbit IgG-BV421 | BioLegend, 406410 | 1/600 | 2.0 | 3.3 |
| donkey anti-rat IgG-AF594 | Invitrogen, A-21209 | 1/300 | 2.0 | 6.6 |
| donkey anti-goat IgG-AF647 | Invitrogen, A-21447 | 1/600 | 2.0 | 3.3 |
| donkey anti-goat IgG-AF549 | Invitrogen, A-11058 | 1/300 | 2.0 | 6.6 |
| donkey anti-rabbit IgG-AF647 | Invitrogen, A-31573 | 1/300 | 2.0 | 6.6 |
| donkey anti-rat IgG-AF488 | Invitrogen, A-21208 | 1/300 | 2.0 | 6.6 |

| **Primary antibody: Western Blot** | **Company and Cat.No.** | **Dilution** | **Starting Conc. Mg/ml** | **Final Conc. Ug/ml** |
| --- | --- | --- | --- | --- |
| rabbit anti-mouse CCL21 | Peprotech, 500-P114 | 1/100 | 0.2 | 2 |
| rabbit anti-human CCL21 | Peprotech, 500-P109 | 1/100 | 0.2 | 2 |
| goat anti-mouse kringle 5 plasminogen | R&D Systems, AF742-SP | 1/1000 | 0.2 | 0.2 |

| **Secondary antibody: Western Blot** | **Company and Cat.No** | **Dilution** | **Starting Conc. Mg/ml** | **Final Conc. Ug/ml** |
| --- | --- | --- | --- | --- |
| donkey anti-rabbit IgG-HRP | Invitrogen, A16035 | 1/1000 | 1 | 1 |
| rabbit anti-goat IgG-HRP | Invitrogen, A27014 | 1/1000 | 1 | 1 |

| **Capture antibody: ELISA** | **Company and Cat.No** | **Dilution** | **Starting Conc. Mg/ml** | **Final Conc. Ug/ml** |
| --- | --- | --- | --- | --- |
| goat anti-mouse CCL21 | R&D Systems, AF457 | 1/100 | 0.2 | 2 |

| **Primary antibody: ELISA** | **Company and Cat.No** | **Dilution** | **Starting Conc. Mg/ml** | **Final Conc. Ug/ml** |
| --- | --- | --- | --- | --- |
| goat anti-mouse CCL21-Biotin | R&D Systems, BAF457 | 1/200 | 0.2 | 1 |
| rat anti-mouse CCL21 | R&D Systems, MAB457-SP | 1/500 | 0.5 | 1 |

| **Secondary antibody: ELISA** | **Company and Cat.No** | **Dilution** | **Starting Conc. Mg/ml** | **Final Conc. Ug/ml** |
| --- | --- | --- | --- | --- |
| streptavidin-HRP | BioLegend, 405210 | 1/500 | 0.5 | 1 |
| donkey anti-rat IgG-HRP | Invitrogen, A-24543 | 1/500 | 1 | 2 |
