# Supplementary material for: uPA-mediated remodeling of CCL21 gradients regulates lymphatic migration of dendritic cells
Source: J Cell Biol. 2026 Jan 27;225(3):e202412190. doi: 10.1083/jcb.202412190 (PMC12839967; doi:10.1083/jcb.202412190)

Collado-Diaz et al.,Compilation of Souce Data (i.e. Western Blots)

General remark: Please note that in most cases, the nitrocellulose membranes were horizonatally cut after blotting and only the lower parts (<30kD) were incubated with antibodies for the detection of CCL21. This way, reagents (i.e. antibodies and ECL solution) could be saved. Moreover, in some cases the upper membrane parts could be used for other W.Blots, e.g. for detecting higher molecular-weight proteins (usch as plasminogen/plasmin).

Figure 3.

3A – Representative Western blot of CCL21 performed on steady-state (CTR) and CHS-inflamed (CHS) murine ear skin protein extracts. Recombinant CCL21 was loaded as a control

Image of the colorimetric ladder (Precision Plus Protein Dual Color, BioRad)

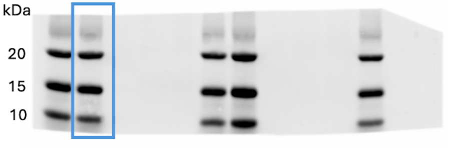

Image of the chemiluminescence signal (same WB imager, same gel position in the imager)

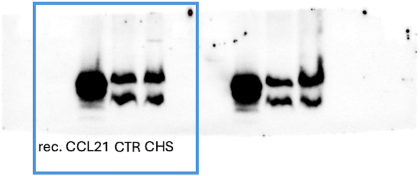

3B – Western blot analysis of recombinant human full-length CCL21 and CCL21-ΔC protein
1 out of 2 experiments is shown

Image of the colorimetric ladder (Precision Plus Protein Dual Color, BioRad)

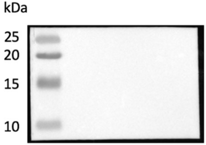

Image of the chemiluminescence signal (same WB imager, same gel position)

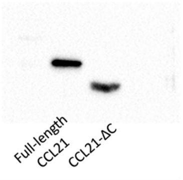

3E – **Schematic depiction of the assay and representative Western blot analysis**

Chemiluminescent signal and colorimetric ladder (Precision Plus Protein Dual Color, BioRad) were imaged separately and overlaid using Image Lab software (BioRad), based on identical gel positioning in the imager.

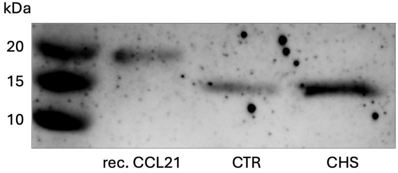

3L – **Western blot analysis of protein extracts of steady-state human skin (CTR)**

Chemiluminescent signal and colorimetric ladder (Precision Plus Protein Dual Color, BioRad) were imaged separately and overlaid using Image Lab software (BioRad), based on identical gel positioning in the imager.

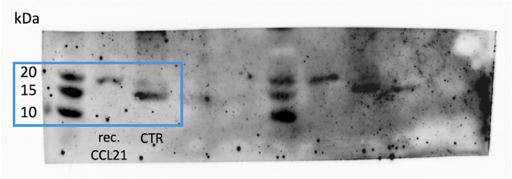

3M – Western blot analysis of protein extracts of Donor-matched steady-state (CTR) and inflamed (INF) human skin from a psoriasis patient

Chemiluminescent signal and colorimetric ladder (Precision Plus Protein Dual Color, BioRad) were imaged separately and overlaid using Image Lab software (BioRad), based on identical gel positioning in the imager.

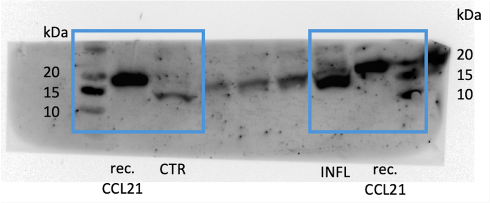

Supplement: SourceData F3 — is the source file for Fig. 3. [file jcb_202412190_sourcedataf3.pdf]
