# Supplementary material for: uPA-mediated remodeling of CCL21 gradients regulates lymphatic migration of dendritic cells
Source: J Cell Biol. 2026 Jan 27;225(3):e202412190. doi: 10.1083/jcb.202412190 (PMC12839967; doi:10.1083/jcb.202412190)

Collado-Diaz et al.,Compilation of Souce Data (i.e. Western Blots)

General remark: Please note that in most cases, the nitrocellulose membranes were horizonatally cut after blotting and only the lower parts (<30kD) were incubated with antibodies for the detection of CCL21. This way, reagents (i.e. antibodies and ECL solution) could be saved. Moreover, in some cases the upper membrane parts could be used for other W.Blots, e.g. for detecting higher molecular-weight proteins (usch as plasminogen/plasmin).

Figure 8

8A - **Representative Western blot performed on tissue protein extracts**

Chemiluminescent signal and colorimetric ladder (Precision Plus Protein Dual Color, BioRad) were imaged separately and overlaid using Image Lab software (BioRad), based on identical gel positioning in the imager.

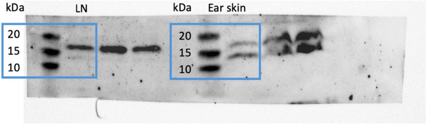

8C – **Representative WB of CCL21 performed on LN extracts, LN eluates and serum**

Image of the colorimetric ladder (Precision Plus Protein Dual Color, BioRad)

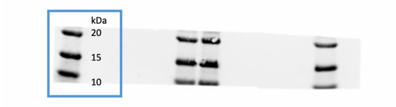

Image of the chemiluminescence signal (same WB imager, same gel position)

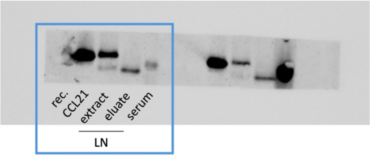

Supplement: SourceData F8 — is the source file for Fig. 8. [file jcb_202412190_sourcedataf8.pdf]
