# Supplementary material for: uPA-mediated remodeling of CCL21 gradients regulates lymphatic migration of dendritic cells
Source: J Cell Biol. 2026 Jan 27;225(3):e202412190. doi: 10.1083/jcb.202412190 (PMC12839967; doi:10.1083/jcb.202412190)

Collado-Diaz et al.,Compilation of Souce Data (i.e. Western Blots)

Sup. Figure 1.

SF1A – WB analysis of human CCL21 cleavage after incubation with recombinant plasmin with a fixed molar ratio of 1:0.08 for increasing times at 37°C, as indicated in the figure

Chemiluminescent signal and colorimetric ladder (Precision Plus Protein Dual Color, BioRad) were imaged separately and overlaid using Image Lab software (BioRad), based on identical gel positioning in the imager.

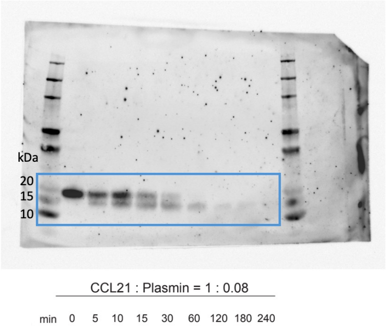

SF1B – WB analysis of murine CCL21 cleavage after incubation with recombinant plasmin with a fixed molar ratio of 1:0.08 for increasing times at 37°C, as indicated in the figure

Chemiluminescent signal and colorimetric ladder (Precision Plus Protein Dual Color, BioRad) were imaged separately and overlaid using Image Lab software (BioRad), based on identical gel positioning in the imager.

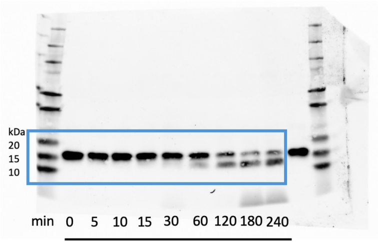

CCL21 : Plasmin = 1:0.08

SF1C – **Dose titration of the plasmin inhibitor C3 to a fixed molar ratio of murine CCL21:plasmin (1:0.08)**

Chemiluminescent signal and colorimetric ladder (Precision Plus Protein Dual Color, BioRad) were imaged separately and overlaid using Image Lab software (BioRad), based on identical gel positioning in the imager.

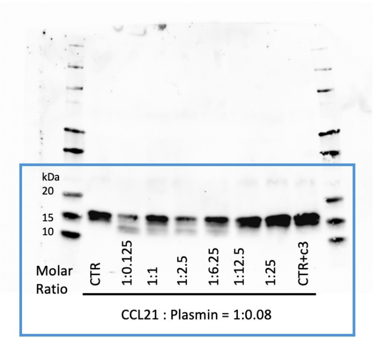

Supplement: SourceData FS1 — is the source file for Fig. S1. [file jcb_202412190_sourcedatafs1.pdf]
