# Supplementary material for: uPA-mediated remodeling of CCL21 gradients regulates lymphatic migration of dendritic cells
Source: J Cell Biol. 2026 Jan 27;225(3):e202412190. doi: 10.1083/jcb.202412190 (PMC12839967; doi:10.1083/jcb.202412190)

Collado-Diaz et al.,Compilation of Souce Data (i.e. Western Blots)

General remark: Please note that in most cases, the nitrocellulose membranes were horizonatally cut after blotting and only the lower parts (<30kD) were incubated with antibodies for the detection of CCL21. This way, reagents (i.e. antibodies and ECL solution) could be saved. Moreover, in some cases the upper membrane parts could be used for other W.Blots, e.g. for detecting higher molecular-weight proteins (usch as plasminogen/plasmin).

Sup. Figure 3.

SF3C – CCL21 cleavage assay performed in presence or absence of LECs and plasminogen (plg), revealing the dependence of CCL21 cleavage on both factors (i.e. LECs and plg)

Image of the colorimetric ladder (Precision Plus Protein Dual Color, BioRad)

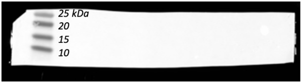

Image of the chemiluminescence signal (same WB imager, same gel position), as shown in SF3C.

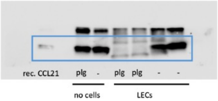

Image below: Brightness and contrast levels were adjusted to enhance visibility of membrane borders.

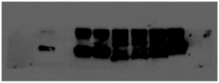

SF3D – **BM-DCs revealing their ability to cleave CCL21 in presence of plg**

Image of the colorimetric ladder (Precision Plus Protein Dual Color, BioRad)

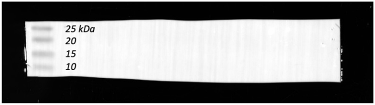

Image of the chemiluminescence signal (same WB imager, same gel position), as shown in SF3D.

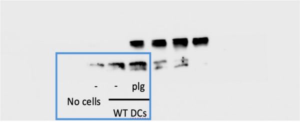

Image below: Brightness and contrast levels were adjusted to enhance visibility of membrane borders.

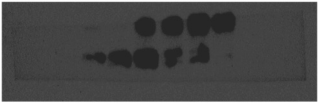

SF3E –keratinocytes revealing their ability to cleave CCL21 in the presence of plg

Chemiluminescent signal and colorimetric ladder (Precision Plus Protein Dual Color, BioRad) were imaged separately and overlaid using Image Lab software (BioRad), based on identical gel positioning in the imager.

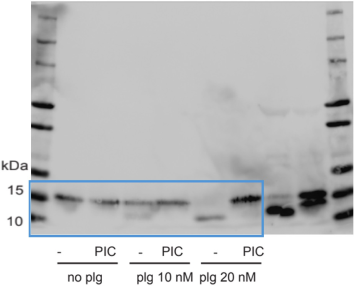

Supplement: SourceData FS3 — is the source file for Fig. S3. [file jcb_202412190_sourcedatafs3.pdf]
